# Molecular Evolution and Protein Structure Variation of *Dkk* Family

**DOI:** 10.3390/genes14101863

**Published:** 2023-09-25

**Authors:** Binhong Wen, Sile Hu, Jun Yin, Jianghong Wu, Wenrui Guo

**Affiliations:** 1 College of Animal Science and Technology, Inner Mongolia Minzu University, Tongliao 028000, China; wenbinhong99@163.com; 2 College of Life Science, Inner Mongolia Minzu University, Tongliao 028000, China; huslee@163.com; 3 College of Animal Science, Inner Mongolia Agricultural University, Hohhot 010018, China; yinjunparis@163.com; 4 College of Veterinary Medicine, Inner Mongolia Agricultural University, Hohhot 010018, China

**Keywords:** *Dkk* gene family, hair follicle, molecular evolution, functional divergence

## Abstract

*Dkks* have inhibitory effects on the Wnt signaling pathway, which is involved in the development of skin and its appendages and the regulation of hair growth. The nucleotide sequences were compared and analyzed to further investigate the relationship between the structure and function of the *Dkk* gene family and vertebrate epidermal hair. The analysis of the molecular evolution of the *Dkk* family revealed that the evolution rate of the genes changed significantly after speciation, with the Aves and Reptilia branches showing accelerated evolution. Additionally, positive selection was observed at specific sites. The tertiary structure of the protein was also predicted. The analysis of the functional divergence of the *Dkk* family revealed that the functional divergence coefficient of each gene was greater than 0, with most of the functional divergence sites were located in the Cys-2 domain and a few in the Cys-1 domain. This suggests that the amino acid and functional divergence sites may play a role in regulating the binding of the *Dkk* family to LRP5/6, and thus affect the inhibition of Wnt signaling, leading to different functions of *Dkk1*, *Dkk2*, and *Dkk4* in the development of skin hair follicles. In addition, the *Dkk* families of Aves and Reptilia may have undergone adaptive evolution and functional divergence.

## 1. Introduction

The *Dkk* gene encodes a protein that acts as an inducer in the head of *Xenopus laevis*, and *Dkk* was found to be a potent antagonist of Wnt signaling [1]. After that, *Dkk* genes were found in vertebrates and invertebrates one after another [2,3,4,5]. The *Dkk* gene family encodes secreted proteins, such as *Dkk1–4* and *soggy* (*Sgy*), which are predominantly found in vertebrates. Four Dkk proteins (Dkk1–4) exist in humans, and all contain two cysteine-rich domains (CRDs), designated CRD1 and CRD2, each of which contains five disulfide bonds [6]. Members of the Dkk protein family can bind to the co-receptor of Wnt, namely low-density lipoprotein receptor-related protein (LRP), and regulate its activity, thus controlling the transduction of Wnt signals. Sgy is a novel secreted protein related to Dkk-3 but which lacks the cysteine-rich domains [2]. The function of *Sgy* is different from that of the other *Dkk* family members. The Dkk family of proteins is known to play a role in regulating the activity of the Wnt signaling pathway, which is involved in the development and regeneration of hair follicles. By modulating the activity of the Wnt signaling pathway, the *Dkk* family can influence the development and periodic regeneration of hair.

Studies have found that the blocking effect of *Dkk1* occurs at the beginning of epidermal morphological changes or cell differentiation caused by molecular signals [7]. A study found that ectopic expression of *Dkk1* in the epidermis leads to defects in tentacles, hair, teeth, and mammary glands [8]. These studies suggest that *Dkk1* negatively regulates hair follicle development and follicle number by antagonizing the canonical Wnt signaling pathway. *Dkk2* inhibits the formation of plantar hair follicles and maintains the normal shape of plantar skin in embryonic development by blocking the Wnt/β-catenin signaling pathway [7]. When *Dkk2* is overexpressed, the activity of the dermal papilla is reduced; when *Dkk2* is knocked out, pulp formation is reduced, both of which lead to delayed feather regeneration [9]. These indicate that knockout and overexpression of *Dkk2* could not maintain the periodic regeneration of hair follicles. *Dkk3* has not been reported to be related to hair follicles. The expression of *Dkk4* was higher in primary hair follicles, but significantly decreased in secondary hair follicles and growing hair follicles [10]. Cui prepared skin-specific *Dkk4* transgenic mice to study the role of *Dkk4* in hair follicle development. In another experiment, the introduction of *Dkk4* into cats and wild-type mice had no effect on primary hair, but the induction of secondary hair and hair follicles was completely prevented [11].

Current research has revealed that the *Dkk* gene family plays a critical role in skin morphology, hair follicle development and growth cycle, and embryonic development of tissue and organs. Vertebrate skin appendages include scales, feathers, and hair; although they look different, they are regulated by the Wnt signaling pathway. This study focused on the functional divergence and molecular evolution of the *Dkk* gene family in the context of skin appendage evolution. In order to gain a better understanding of the genetic variation and evolutionary relationship of the *Dkk* gene family in vertebrates, we retrieved the coding region sequences of *Dkk* gene family members from the Genebank database on the NCBI website for a selection of representative species. Because the structure and function of *sgy* is different from that of *Dkk1–4*, we only analyze *Dkk1–4*. The gene sequences of *Dkk1–4* were then analyzed using molecular evolution techniques to gain insights into their genetic variation and evolutionary relationship. Additionally, the protein structures of these genes were also analyzed to gain further insights into their function and role in vertebrate evolution.

## 2. Results

### 2.1. Sequence Alignment and Phylogenetic Analyses

**Figure 1:**
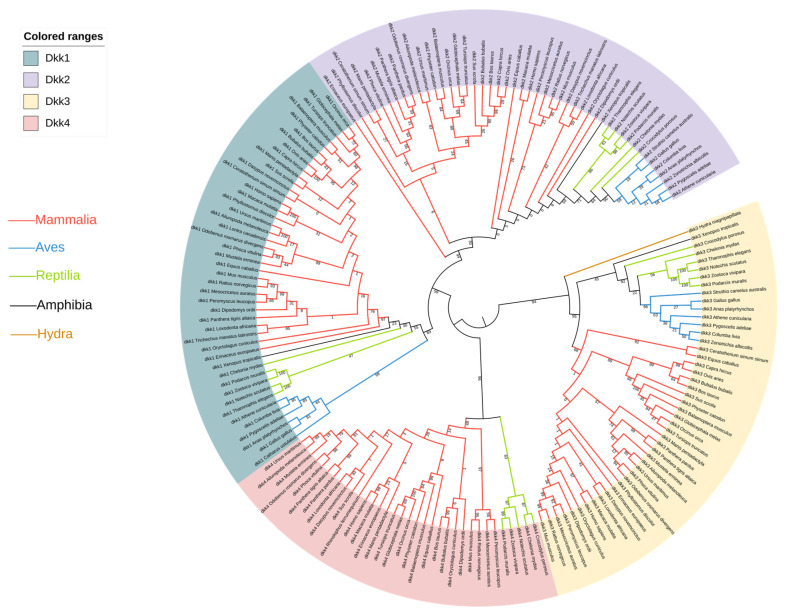
Phylogenetic tree of *Dkk* gene family. The numbers on the evolutionary tree are bootstrap values.

**Figure 2:**
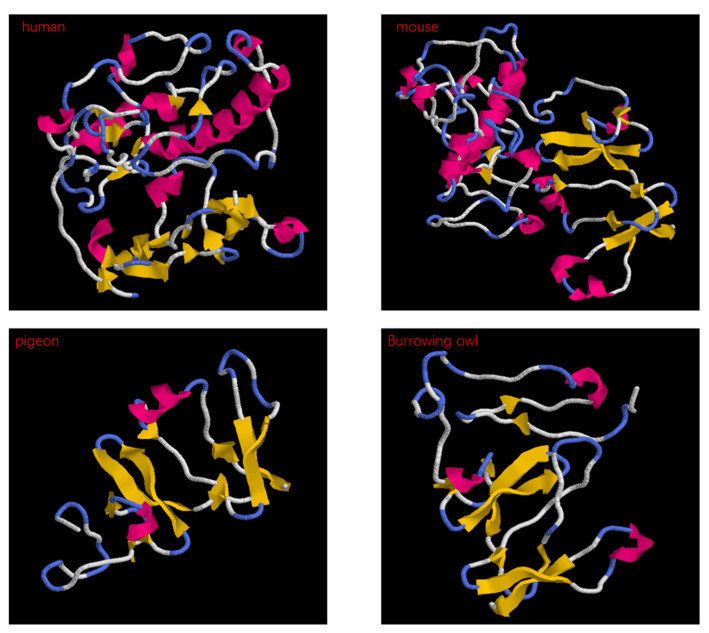
Protein structure of human, mouse, pigeon, and burrowing owl *Dkk1*. Deep red represents α-helix, yellow represents β-fold, light blue represents coil, and white represents other residues.

To study the phylogenetic relationship between the different genes, a phylogenetic tree was constructed using the Maximum Likelihood method. From the topology of the tree, it can be seen that *Dkk1*, *Dkk2*, and *Dkk4* all originated from *Dkk3* (Figure 1). Multiple sequence alignments were performed for the *Dkk1–4* genes. There are obvious differences in the gene sequence of *Dkk1* in vertebrates; Aves lack 10 amino acids in the middle region (Figure S1A). This study also found that the protein structure in Aves is different from that of humans and mice (Figure 2). From the tertiary structure of proteins, it can be observed that avian protein sequences are shorter and lack a significant amount of α-helices. In the multiple sequence alignment of *Dkk2*, there were differences between Aves and Reptilia: 14 amino acids were inserted in Aves and Reptilia (Figure S1B). In the multiple sequence alignment of *Dkk3*, there are differences among Aves, Reptilia, Anura, and Chiroptera; 11 amino acids were inserted in Aves and Reptilia (Figure S1C). The results of the multiple sequence alignment of *Dkk4* showed no significant differences.

### 2.2. Variation in Molecular Evolution Rate of Dkk

To examine the changes in selection pressure on the four genes of the *Dkk* gene family during the evolution of vertebrates, we calculated the *ω* values using the model M0 in PAML (Phylogenetic Analysis by Maximum Likelihood): *ω_Dkk1_* = 0.10793, *ω_Dkk2_* = 0.06302, *ω_Dkk3_* = 0.20621, and *ω_Dkk4_* = 0.26750. The results show that the synonymous substitution rate is much higher than the non-synonymous substitution rate, indicating that the entire *Dkk* gene family has generally undergone purifying selection during the evolution of vertebrates, showing the functional conservation of this gene family.

Although the overall *Dkk* gene sequence was strongly purified and selected, it could not be ruled out that some amino acids at specific sites were under positive selection. Therefore, we used Site models (M3 vs. M0 and M2 vs. M1) to detect the selection pressure at all sites. The LRT difference between M3 and M0 of *Dkk1* was significant (2Δl = 10858.42, df = 4, *p* < 0.001), indicating that discrete selection pressures were applied to different sites of *Dkk1*, but none of the sites were in a positive selection state. The LRT between M1 and M2 of *Dkk1* does not support the hypothesis that model M2 is superior to model M1 (2Δl = 0, df = 2, *p* > 0.05), and there is no positive selection site. The results of each model are shown in Table S2. The results showed that all *Dkk* genes were under purifying selection, and that no positive sites were found.

Although all four genes showed purifying selection, a free-ratio model was developed in order to better reflect the selection pressure of different species in evolution. The results showed that in *Dkk1*, the *ω* value of Reptilia (*ω* = 21.85) was significantly higher than that of other branches. In *Dkk3*, Aves and Reptilia have *ω* = 3.04. However, in Aves species, *ω* = 999 for the *Dkk1* and *Dkk3* genes. In *Dkk2* and *Dkk4*, the *ω*-value of the branch of Pholidota and Carnivora was significantly higher than that of the other branches (*ω* > 1). A *ω* ratio significantly greater than one is a convincing indicator of positive selection. These results suggest that positive selection may have played a potential role in the early evolution of Reptilia, Aves, Pholidota, and Carnivora.

To further determine if any amino acid sites in the *Dkk* gene family’s accelerated evolution branch are under selection pressure, we ran Model A (Model = 2, NSsites = 2) in the branch site model, which takes into account not only the *ω* value between sites but also the *ω* value between branches. The test results (Table 1) are all based on the probability calculated by the BEB method (*: *p* > 95%; **: *p* > 99%).

### 2.3. Dkks Functional Divergence Analysis Results

According to the previously constructed species evolution tree, the vertebrates were divided into three groups: A for Mammalia, B for Reptilia, and C for Aves (Table 2 and Table 3). Most of these functional divergence sites are distributed in the Cys-2 domain, and very few are distributed in the Cys-1 domain. In the *Dkk* gene family, *Dkk2*, *Dkk3*, and *Dkk4* had obvious type I functional divergence; the value of *θ_I_* was between 0.23 and 0.63, while the *θ_I_* coefficient of *Dkk1* was relatively small. The *θ_II_* coefficients of *Dkk1–4* are all relatively small (Table 2 and Table 3).

### 2.4. Dkk Protein Analysis Results

To infer structure–function correlations, the sequences of the positive selection sites were detected using the PAML software and are based on the human amino acid sequence. This is because some amino acids will be deleted when the PAML software is running, so it is necessary to determine the position of the detected amino acid position in the complete amino acid sequence. The pictures of the involved sites are shown in Figure S3. We found that there was a change in the protein sequence of *Homo sapiens* (255S) and *Anas platyrhynchos* (223P) *Dkk1*, and the protein structure changed from a turn to a coil. This amino acid site is located in the Cys-2 domain. In *Dkk2*, the amino acid site (27V) of *H. sapiens* and the corresponding amino acid site of *Manis pentadactyla* (86M) changed, and the protein structure changed from a turn to a coil. This amino acid site is located in the Cys-1 domain. In *Dkk3*, the amino acid site (264R) of *H. sapiens* and the corresponding amino acid site of *Zonotrichia albicollis* (209L) changed, and the protein structure changed from a turn to a coil. In *Dkk4*, the amino acid site (132K) of *H. sapiens* and the corresponding amino acid site of *Podarcis muralis* (131Q) changed, and the protein structure changed from a coil to a turn.

## 3. Discussion

In this paper, we investigated the evolutionary relationship of Dkk proteins in vertebrates. We discuss the nature of *Dkk*’s interactions with its partner Krm1 and the E3E4 region of LRP5/6, which have been widely established, and the functional divergence of Dkk proteins in vertebrate evolution, which has not been reported. We also explore the surprising lack of understanding of the *Dkk* family in terms of molecular evolution. Our findings provide insight into the evolution of Dkk proteins and their role in Wnt signaling.

In the multiple sequence alignment, *Dkk1*, *Dkk2*, and *Dkk3* showed obvious differences. The inserted amino acids have an irregular curl in the protein structure. The insertion of these amino acids may cause the *Dkk* genes to differ in skin phenotype between different species. Through the construction of a phylogenetic tree, the phylogenetic relationship between species or genes can be displayed clearly. *Dkk1*, *Dkk2*, and *Dkk4* all originated from *Dkk3*. A study confirmed that vertebrate *Dkk*-*1*, *2*, and *4* may have originated from a common ancestor gene of *Dkk3* [12]. But, in another study, *Dkk3* appears to be a divergent member of the *Dkk* family [13]. The origin of the *Dkk* family is currently debated, but our results confirm that they originate from *Dkk3*. Due to structural differences, *Dkk3* proteins exhibit biological characteristics that are different from those of the other family members. Most of the reports on *Dkk3* gene are closely related to the occurrence, development, metastasis, and prognosis of common tumors [14]. *Dkk1*, *Dkk2*, and *Dkk4* have all been reported to be involved in hair follicle development. The *Dkk* family may play an important role in hair follicle variation in vertebrates. To test this hypothesis, we used CodeML estimates of synonymous and non-synonymous substitutions.

The results of the M0 model indicate that the *Dkk* gene family is under purifying selection in most vertebrates. The M0 model indicates that this gene family has important functions. In addition, the free-ratio model evolution studies have shown that, in vertebrates, the selection pressure of the *Dkk* family changes. The adaptive evolution of the four genes occurred primarily in single branches of the phylogenetic tree, including Reptilia, Aves, Cetaceans, Lepidoptera, and Carnivora. For Reptilia and Aves *Dkk1* and *Dkk3* genes, *ω* > 1. An *ω* ratio significantly higher than 1 is convincing evidence of diversification [15]. Aves *Dkk1* and *Dkk3* showed the strongest positive selection signal (*ω*  =  999) based on the branch mode. Reptilian scales and avian feathers are considered homologous structures [16]. The complex topology of bird feathers may be responsible for the accelerated evolution of birds during feather development. The topology of bird feathers is more complex than that of reptile scales [17]. Feathers consist of many tiny structures that form complex interactions and liaisons between each other. This complex structure allows birds to be more adaptable in terms of flight and protecting themselves. However, in the evolution of vertebrates, the hair phenotypes of Reptilia and Aves are unique. Given that diversity in hair development can occur through multiple pathways, this lack of a parallel signature is perhaps not surprising [18]. The results of the Branch site model of the *Dkk* gene family showed that there were adaptive evolution and purifying selection sites in Reptilia in *Dkk1*, *Dkk3*, and *Dkk4*. There are also purifying selection sites in birds in *Dkk1* and *Dkk3*. Moreover, the amino acid sites we found almost all existed in the CRD. It was found that the CRD domain is both necessary and sufficient for the binding to Wnt [19,20]. However, the Wnt signaling pathway plays an important role in many genes or signaling pathways that regulate hair follicle growth and development [21,22].

To further determine whether the *Dkk* gene family had functional divergence among species, family members were tested for type I and type II functional divergence. The degree of functional divergence of type I is greater than that of type II. This indicates that the functional constraints between replicative genes have changed [23]. Our results also proved that the *Dkk* gene family underwent accelerated evolution during species evolution, and some residues may have undergone functional restriction changes after speciation. Mammals, Aves, and Reptilia have different hair phenotypes, which may cause the genes to have different functions. At the same time, one purifying selection site (281V) in the *Dkk3* gene was also identified as functional divergence site by the pressure selection analysis.

**Figure 3:**
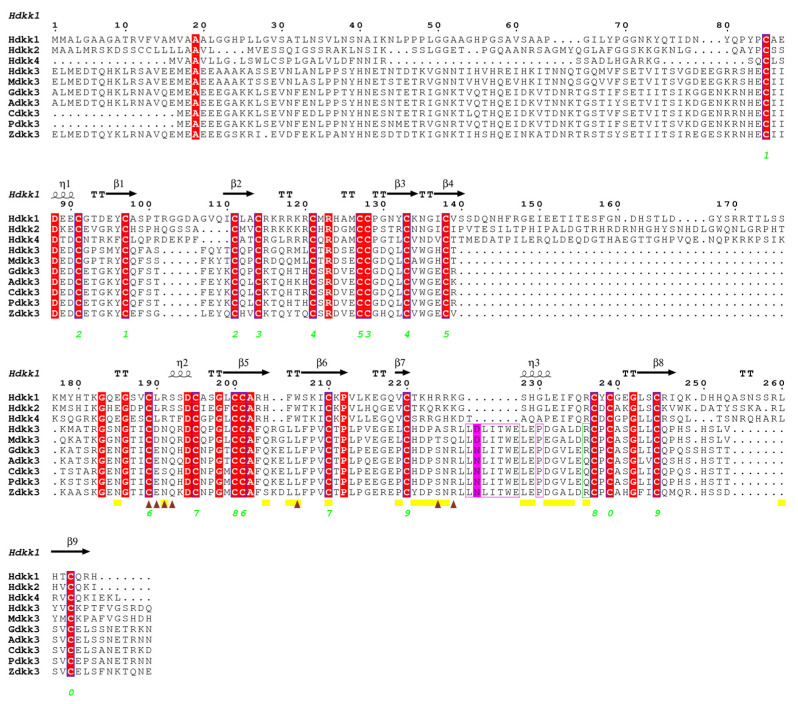
*Dkk* multiple sequence alignment. Highly similar residues are colored in red and framed in blue. The green number indicates the disulfide bond. The yellow box at the bottom of the sequence represents the residue involved in the interaction between *Dkk1* and LRP6. The brown triangle is the residue involved in the interaction between *Dkk1* and Kremen proteins. Seven amino acids (L249-E255 in human *Dkk3*, LDLITWE) and P258 are represented in a purple box. In the purple box, the black letter is the site of a *Dkk3* mutation (D250N). The green box is the Arg264Gln of *Dkk3* gene. Abbreviations: H = *H. sapiens*, M = *Mus musculus*, G = *G. gallus*, A = *Anas platyrhynchos*, C = *Columba livia*, P = *Pygoscelis adeliae*, and Z = *Z. vivipara*.

**Figure 4:**
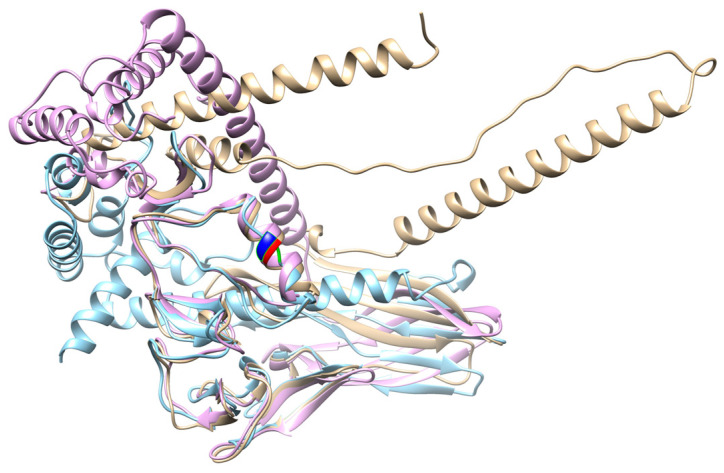
Overlay structure of *Dkk3* proteins from three species. Species: *H. sapiens* (brown), *Zootoca vivipara* (light blue), and *G. gallus* (pink). Red, green, and dark blue represent the relative positions of the D250N mutation. The secondary structure of 250N in *Z. vivipara* is a random coil.

Most of the purifying selection sites and functional divergence sites screened in this study are in the middle and downstream regions, and some of them are in the Cys-2 domain. *Dkk1*, *Dkk2*, and *Dkk4* have been identified as effective inhibitors of Wnt signaling and bind to the Wnt coreceptor LRP5/6 [24,25,26]. However, some studies have found that CRD2 is essential for suppressing Wnt signals [27]. The binding site of *Dkk1* CRD2 to LRP5/6 has been reported [28,29]. The binding sites of *Dkk1* and Kremen1 have also been reported [30,31]. Neutral sites were found near the binding sites. Mohammadpour performed in silico analyses and established that *Dkk3*, similar to other *Dkk* family members, can bind to the third PE pair of LRP5/6 through its CRD2 [32]. In another in silico study, Fujii reported that the insertion of seven amino acids (L249-E255 in human *Dkk3*) and P258 reduced the binding affinity between *DKK3* and LRP5/6 [33]. Interestingly, the D250 residue in the Dkk3 protein sequence was mutated to N in Aves, Reptilia, and Amphibia (Figure 3). This mutation leads to a decrease in amino acid hydrophilicity. Comparing the tertiary structures of three species (*H. sapiens*, *Zootoca vivipara*, and *Gallus gallus*), the secondary structure of D250N changed from a turn to a random coil (Figure 4). When the hydrophilicity of an amino acid is weakened, it may have an effect on the structure and function of the protein [34].

A study designed and improved several small peptides based on the LRP6-binding site of the CRD2 of *Dkk3* [35]. These peptides were highly capable of binding to LRP6 in silico, and may prevent the formation of an active Wnt-LRP6-Fz complex [35]. In the experiment conducted by Poorebrahim, several small peptides did not alter 264R, an amino acid that forms a salt bridge with Asp811 of LRP6. In our experiment, we found a mutation in 264R in the Aves species, where Arg was replaced by Gln, resulting in decreased hydrophilicity (Figure 3). In the protein structure, two amino acid residues with opposite charges form an ion pair; when the distance between the charged groups of the two amino acid side chains in the ion pair (that is, any oxygen atom in the negatively charged residue carboxylate and the positively charged residue side or the distance between any nitrogen atom in the chain) is less than 4 Å, the ion pair is considered a salt bridge [36,37]. Gln is a polar uncharged residue. The Arg264Gln substitution would abolish a salt bridge with Asp811 in LRP6. The selected amino acids found in CRD2 may affect the binding of the *Dkk* gene family to LRP5/6, thus affecting the inhibition mechanism of Wnt signals and making *Dkk1*, *Dkk2*, and *Dkk4* show different functions in hair follicle development. Vertebrate habitats range from the deepest parts of the ocean to the highest peaks of mountain ranges, from the tropics to the Arctic. The environmental variations trigger divergent natural selection, leading to the emergence of niche specialists [27]. The living environment of Aves and Reptilia may also cause these genes to differentiate in function during the evolutionary process.

## 4. Conclusions

*Dkk1–4* all underwent accelerated evolution and purifying sites were detected. The changes in the *Dkk* gene family in vertebrates under selection pressure and functional divergence were tested. However, the evolution rates of purifying selection sites and functional divergence sites are different. These amino acid sites will affect the tertiary structure of proteins and make genes differentiate functionally. The current study shows that the *Dkk* gene family underwent changes in selection patterns during vertebrate evolution and may have acquired additional functional constraints in different branches.

## 5. Materials and Methods

### 5.1. Sequence Acquisition

Sequence data of the CDS of the *Dkk1–4* genes of different species were retrieved from the GenBank database. For the *Dkk1* gene, a total of 45 species were selected, 47 species were selected for the *Dkk2* gene, 47 species for the *Dkk3* gene, and 34 species for the *Dkk4* gene (Table S1). These data were then used to analyze the genetic variation and evolutionary relationship of the *Dkk* gene family in vertebrates. *Hydra magnipapillata* is an invertebrate. We chose *Hydra* as an outgroup because we wanted to study the origin of the *Dkk* gene family. The *Dkk* gene family has been found in vertebrates and some invertebrate phyla but *Dkk4* appears to only be present in mammals and Reptilia.

### 5.2. Nucleotide Sequence Analysis

Using MEGA-X [38], the Maximum Likelihood tree of the amino acid sequences of the *Dkk* gene family of vertebrates was constructed, and the confidence value of each branch was calculated with 500 repetitions of the Bootstrap test. Additionally, the nucleotide sequences of the *Dkk1–4* genes of the above species were aligned using the ClustaW method using MEGA-X. Itol (http://itol.embl.de/ accessed on 1 May 2023) was used to beautify the evolutionary trees. The multiple sequence alignment diagram of the amino acid sequence was generated using ESPript 3 (https://espript.ibcp.fr/ESPript/ESPript/index.php accessed on 20 May 2023).

### 5.3. Molecular Evolution Analysis

To detect changes in the selection pressure of four genes in the *Dkk* gene family during vertebrate evolution, the CodeML program in PAMLX [39] was used to calculate the ω (dN/dS) values of the *Dkk1–4* sequences, where dN represents the rate of non-synonymous substitutions, and dS represents the rate of synonymous substitutions. dN/dS < 1 means purifying selection, dN/dS = 1 means neutral evolution, and dN/dS > 1 means positive Darwinian selection.

To test whether there is positive selection at specific amino acid sites, we compared four models: M3 vs. M0 and M2 vs. M1. The rates along specific branches of the tree were estimated, and the topology is based on published studies [40,41], as shown in Figure S2. We performed a one-ratio model for each gene separately, which assumes equal ω values for all branches of the phylogenetic tree [42]. Secondly, a free-ratio model was developed, which assumes that the ω value is different for all branches in the phylogenetic tree [43], and analyzes the selection pressure of this gene at the branch level of different species. Finally, Model A of the branch site model (Model = 2, NSsites = 2) was developed, which analyzes the amino acid sites on the branch with high selection pressure where members of the *Dkk* gene family are positively selected [43]. All species comparisons were made using *H. sapiens* as a reference.

To determine whether the *Dkk* gene family has functional divergence among the various branches of the phylogenetic tree, DIVERGE3.0 [44] was used to test the functional divergence of each gene. Functional divergence was measured by the functional divergence coefficient (θ). The value of θ is between 0 and 1, and the closer the value of θ is to 1, the more significant the functional divergence of the gene clusters is. At least three species are needed to form a group in species functional divergence analysis. Among amniotes, stem reptiles were basal to extant reptiles, birds, and mammals [45]. In this study, it was found that there is significant divergence between Reptilia and Aves, and vertebrates were divided into three groups, namely, A for Mammalia, B for Reptilia, and C for Aves. Any two groups were compared and analyzed, and type I and type II functional divergences were analyzed.

### 5.4. Protein Analysis

To infer structure–function correlations, positively selected amino acid residues were mapped to the three-dimensional structure of *Dkk* proteins. The *H. sapiens* protein structure was from the AlphaFold database. However, the tertiary structures of proteins of the *Dkk* gene family were not available in protein databases (https://espript.ibcp.fr/ESPript/ESPript/index.php accessed on 15 Jan 2023). The tertiary structures of *Dkk1*, *Dkk2*, *Dkk3*, and *Dkk4* proteins from different species were predicted by Robetta (https://robetta.bakerlab.org/ accessed on 16 Apr 2023). We used Rasmol to display the protein tertiary structure of *Dkks*. Models were superimposed using MatchMaker from CHIMERA 1.16 (https://www.cgl.ucsf.edu/chimera/ accessed on 25 May 2023).

## Figures and Tables

**Table 1:** Results of branch site model. *: *p* > 95%; **: *p* > 99%.

Gene	Foreground Branch	Possible Positive Selection Sites
*Dkk1*	Aves	17 H 0.997 **, 75 Y 0.998 **, 91 S 0.981 *
	Aves and Reptilia	7 H 0.979, 75 Y 1.000 **
*Dkk2*	Lepidoptera (pangolin)	27 V 0.998 **
*Dkk3*	Aves and Reptilia	24 R 0.997 **, 41 R 0.998 **, 52 P 1.000 **, 58 V 0.999 **
*Dkk4*	Reptilia	103 S 0.975 *, 105 K 0.970 *, 108 Q 0.961 *

**Table 2:** Functional divergence of type I *Dkk* gene family.

Gene	Clusters	*θ_I_*	MFE SE	Possible Positive Selection Sites (Qk > 0.90)
*Dkk1*	A/B	−0.064754	0.145614	
	A/C	0.143104	0.155194	
	B/C	0.068558	0.216823	
*Dkk2*	A/B	0.239265	0.140598	
	A/C	0.403401	0.100621	171G
	B/C	0.514811	0.144675	
*Dkk3*	A/B	0.427149	0.301757	367T, 377H, 410R, 414H, 416L, 423T, 443V, 450G, 495E
	A/C	0.630269	0.134886	399R, 407L, 411H
	B/C	0.623537	0.348995	399R
*Dkk4*	A/B	0.332477	0.090430	185Q

**Table 3:** Functional divergence of type II *Dkk* gene family.

Gene	Clusters	*θ_II_*	MFE SE	Possible Positive Selection Sites (Qk > 0.90)
*Dkk1*	A/B	−0.038624	0.086041	
	A/C	0.059911	0.081074	263Y, 268Q, 271S, 312S, 324G, 328S, 341N, 342S
	B/C	0.010027	0.088840	252Y, 261K, 324G
*Dkk2*	A/B	0.035892	0.052704	165H, 188H
	A/C	0.097338	0.043583	36A, 38L, 44S, 46G, 92Q, 97S, 98S, 114G, 161D, 165H, 166R, 170H, 174S, 176N, 177H, 188H, 212F, 266Y, 270A
	B/C	−0.020593	0.043545	
*Dkk3*	A/B	0.141160	0.144634	360G, 371V, 373G, 375L, 377H, 380A, 385D, 404S, 410P, 411H, 416L, 417V, 418Y, 421K, 422P, 423T, 443V, 444G, 446R, 447D, 450G, 472D, 477G, 484E, 487R, 488Q, 492D, 495E, 496R, 507P, 516G
	A/C	0.225194	0.105301	360G, 371V, 375L, 380A, 381S, 385D, 398D, 411H, 416L, 417V, 418Y, 421K, 422P, 423T, 443V, 444G, 447D, 448Q, 450G, 471P, 472D, 484E, 488Q, 496R, 507P, 516G
	B/C	−0.016133	0.132199	373G, 471P, 477G
*Dkk4*	A/B	−0.081373	0.135288	

## Data Availability

All data generated or analyzed during this study shall be made available upon reasonable request.

## Supplementary Materials

The following supporting information can be downloaded at: https://www.mdpi.com/article/10.3390/genes14101863/s1, Figure S1: Multiple sequence alignment. (A) In *Dkk1*, Aves lack 10 amino acids in the middle region, while 10 amino acids are inserted in other vertebrates. (B) In *Dkk2*, mammals and Anura lacked 13 amino acids in the middle segment, and 14 amino acids were inserted in Aves and Reptilia. (C) In *Dkk3*, 12 amino acids were inserted in *Xenopus tropicalis* and *Phyllostomus discolor* and Amphibia, and 11 amino acids were inserted in Aves and Reptilia; Figure S2: Phylogenetic tree of Dkk gene family. (A) The type I (θI) and type II (θII) functional divergences of the *Dkk1* gene were estimated between mammals and Reptilia, mammals and Aves, and Reptilia and Aves. (B) The type I (θI) and type II (θII) functional divergences of the *Dkk2* gene were estimated between mammals and Reptilia, mammals and Aves, and Reptilia and Aves. (C) The type I (θI) and type II (θII) functional divergences of the *Dkk3* gene were estimated between mammals and Reptilia, mammals and Aves, and Reptilia and Aves. (D) The type I (θI) and type II (θII) functional divergences of the *Dkk4* gene were estimated between mammals and Reptilia; Figure S3. The amino acids under positive selection are mapped onto the 3D structure of the protein. (A) In *Dkk1*, a is *H. sapiens*, the positively selected amino acid (255S) is shown in green, and the structure is the corner. b is Anas platyrhynchos, the corresponding amino acid (223P) is shown in green, and the structure is a coil. (B) In *Dkk2*, a is *H. sapiens*, the positively selected amino acid (86V) is shown in green, and the structure is the corner. b is Manis pentadactyla, the corresponding amino acid (86M) is shown in green, and the structure is a coil. (C) In *Dkk3*, a is *H. sapiens*, the positively selected amino acid (264R) is shown in green, and the structure is the corner. b is Zonotrichia albicollis, the corresponding amino acid (209L) is shown in green, and the structure is not a regular coil. (D) In *Dkk4*, a is *H. sapiens*, the positively selected amino acid (132K) is shown in green, and the structure is an irregular coil. b is Podarcis muralis, the corresponding amino acid (131Q) is shown in green, and the structure is the corner; Table S1: Species, scientific names, and accession numbers of the *Dkk* gene family; Table S2: Parameters and results of evolutionary model of *Dkk* gene family.
