# Supplementary material for: Molecular Evolution and Protein Structure Variation of Dkk Family
Source: Genes (Basel). 2023 Sep 25;14(10):1863. doi: 10.3390/genes14101863 (PMC10606412; doi:10.3390/genes14101863)
Supplement: Supplementary file 1 [file genes-14-01863-s001.zip › genes-2601686-supplementary.pdf]

Molecular Evolution and Protein Structure Variation of *Dkk* Family

Binhong Wen[1], Husile Gong[2], Jun Yin[3], Jianghong Wu[1*], WenRui Guo[4]

1. College of Animal Science and Technology, Inner Mongolia Minzu University, Tongliao, 028000, China
2. College of Life Science, Inner Mongolia Minzu University, Tongliao, 028000, China
3. College of Animal Science, Inner Mongolia Agricultural University, Hohhot,01001, China
4. College of Veterinary Medicine Inner Mongolia Agricultural University, Hohhot, 010018, China

* Corresponding authors: Jianghong Wu (wujianghonglong@126.com, College of Animal Science and Technology, Inner Mongolia Minzu University, Tongliao, 028000, China)

Email addresses

Binhong Wen wenbinhong99@163.com

Husile Gong huslee@163.com

Jun Yin yinjunparis@163.com

Jianghong Wu wujianghonglong@126.com

WenRui Guo gwrui@163.com

Table S1 Species, scientific names and accession numbers of the *Dkk* gene family

Gene	Species name	Accession number
Dkk1	*Homo sapiens*	NM_012242
	Mus musculus	NM_010051
	Oryctolagus cuniculus	NM_001082737
	Bos taurus	NM_001205544
	Ovis aries	XM_012102454
	Loxodonta africana	NM_001280880
	Equus caballus	NM_001267802
	Manis pentadactyla	XM_036924093
	Dasypus novemcinctus	XM_004462400
	Tursiops truncatus	XM_004325200
	Panthera tigris altaica	XM_007098121
	Macaca mulatta	NM_001260525
	Phyllostomus discolor	XM_028512165
	Orcinus orca	XM_004286283
	Balaenoptera musculus	XM_036828886
	Physeter catodon	XM_007110600
	Globicephala melas	XM_030864963
	Lontra canadensis	XM_032882999
	Capra hircus	XM_005698161
	Trichechus manatus latirostris	XM_004369892
	Erinaceus europaeus	XM_007520747
	Ceratotherium simum simum	XM_004440005
	Odobenus rosmarus divergens	XM_004408626

	Phoca vitulina	XM_032430367
	Mesocricetus auratus	XM_005063697
	Rattus norvegicus	NM_001106350
	Mustela erminea	XM_032313600
	Peromyscus leucopus	XM_028895210
	Dipodomys ordii	XM_013017131
	Ursus maritimus	XM_008689579
	Ailuropoda melanoleuca	NM_001304925
	Bubalus bubalis	XM_006072352
	Panthera pardus	XM_019430081
	Xenopus tropicalis	NM_001016283
	Anas platyrhynchos	XM_027460718
	Gallus gallus	XM_015278709
	Athene cunicularia	XM_026851768
	Columba livia	XM_005505220
	Pygoscelis adeliae	XM_009332773
	Catharus ustulatus	XM_033066438
	Podarcis muralis	XM_028729659
	Zootoca vivipara	XM_035138222
	Chelonia mydas	XM_007071596
	Notechis scutatus	XM_026680111
	Thamnophis elegans	XM_032231678
Dkk2	*Homo sapiens*	NM_014421
	Mus musculus	NM_020265
	Oryctolagus cuniculus	XM_002717198
	Bos taurus	NM_001082615
	Ovis aries	XM_004009640
	Capra hircus	XM_005681333
	Loxodonta africana	XM_003410387
	Trichechus manatus latirostris	XM_004380218
	Dasypus novemcinctus	XM_004476199
	Equus caballus	XM_001503589
	Tursiops truncatus	XM_019927773
	Panthera tigris altaica	XM_007080813
	Macaca mulatta	XM_001085254
	Phyllostomus discolor	XM_028507057
	Orcinus orca	XM_004269601
	Balaenoptera musculus	XM_036854041
	Physeter catodon	XM_024119509
	Globicephala melas	XM_030864119
	Manis pentadactyla	XM_036920940
	Erinaceus europaeus	XM_007531232
	Ceratotherium simum simum	XM_004426603
	Odobenus rosmarus divergens	XM_004401893

	Phoca vitulina	XM_032400962
	Sus scrofa	XM_003129269
	Mesocricetus auratus	XM_005081990
	Rattus norvegicus	NM_001106472
	Mustela erminea	XM_032333084
	Peromyscus leucopus	XM_028856981
	Dipodomys ordii	XM_013023723
	Ursus maritimus	XM_008689401
	Ailuropoda melanoleuca	XM_002916941
	Bubalus bubalis	XM_006051404
	Panthera pardus	XM_019416677
	Xenopus tropicalis	XM_002940290
	Zonotrichia albicollis	XM_005484690
	Anas platyrhynchos	XM_021272669
	Gallus gallus	XM_420494
	Athene cunicularia	XM_026848166
	Struthio camelus australis	XM_009689378
	Columba livia	XM_021283065
	Pygoscelis adeliae	XM_009327057
	Podarcis muralis	XM_028744214
	Zootoca vivipara	XM_035113453
	Chelonia mydas	XM_007054398
	Notechis scutatus	XM_026668664
	Thamnophis elegans	XM_032224362
	Crocodylus porosus	XM_019534137
Dkk3	*Hydra magnipapillata*	AY332609
	Homo sapiens	NM_001018057
	Mus musculus	NM_001360257
	Oryctolagus cuniculus	XM_008266255
	Bos taurus	NM_001100306
	Ovis aries	XM_027979387
	Capra hircus	XM_018059550
	Loxodonta africana	XM_023550844
	Manis pentadactyla	XM_036918028
	Dasypus novemcinctus	XM_012522734
	Equus caballus	XM_023646049
	Tursiops truncatus	XM_019948202
	Panthera tigris altaica	XM_015537970
	Macaca mulatta	XM_028832590
	Phyllostomus discolor	XM_028515585
	Orcinus orca	XM_004279179
	Globicephala melas	XM_030831410
	Balaenoptera musculus	XM_036861902
	Physeter catodon	XM_024118577

	Erinaceus europaeus	XM_007521154
	Ceratotherium simum simum	XM_004418440
	Odobenus rosmarus divergens	XM_004394593
	Phoca vitulina	XM_032427021
	Sus scrofa	NM_001039749
	Mesocricetus auratus	XM_013117477
	Rattus norvegicus	NM_138519
	Mustela erminea	XM_032358081
	Peromyscus leucopus	XM_028875843
	Dipodomys ordii	XM_013028673
	Ursus maritimus	XM_008711972
	Ailuropoda melanoleuca	XM_002925297
	Bubalus bubalis	XM_006055228
	Panthera pardus	XM_019456330
	Xenopus tropicalis	NM_001123476
	Zonotrichia albicollis	XM_005492066
	Anas platyrhynchos	XM_027461217
	Gallus gallus	NM_205125
	Athene cunicularia	XM_026850892
	Struthio camelus australis	XM_009687258
	Columba livia	XM_005499299
	Pygoscelis adeliae	XM_009334472
	Podarcis muralis	XM_028705427
	Zootoca vivipara	XM_035119042
	Chelonia mydas	XM_037899824
	Notechis scutatus	XM_026664838
	Thamnophis elegans	XM_032224195
	Crocodylus porosus	XM_019552185
Dkk4	*Homo sapiens*	NM_014420
	Mus musculus	NM_145592
	Oryctolagus cuniculus	XM_002720793
	Bos taurus	XM_015460894
	Loxodonta africana	XM_003412504
	Manis pentadactyla	XM_036877486
	Dasypus novemcinctus	XM_004481696
	Equus caballus	XM_001915312
	Tursiops truncatus	XM_033848813
	Panthera tigris altaica	XM_007097034
	Macaca mulatta	XM_001097758
	Rhinolophus ferrumequinum	XM_033105033
	Orcinus orca	XM_004285084
	Globicephala melas	XM_030829797
	Balaenoptera musculus	XM_036838881
	Physeter catodon	XM_007127123

Erinaceus europaeus	XM_007522781
Odobenus rosmarus divergens	XM_004415734
Phoca vitulina	XM_032413257
Sus scrofa	XM_021077993
Mesocricetus auratus	XM_005066535
Rattus norvegicus	NM_001109332
Peromyscus leucopus	XM_028887817
Dipodomys ordii	XM_013024410
Ursus maritimus	XM_008706289
Ailuropoda melanoleuca	XM_019798688
Bubalus bubalis	XM_006075711
Panthera pardu	XM_019440676
Xenopus tropicalis	NM_001123476
Podarcis muralis	XM_028741144
Zootoca vivipara	XM_035118651
Chelonia mydas	XM_007064183
Notechis scutatus	XM_026684761
Crocodylus porosus	XM_019546036

Table S2 parameters and results of evolutionary model of *Dkk* gene family

Gene	Model	np	InL	Parameter	$2\Delta l$	Positive selection site
Dkk1	Free-ratio	177	-3570.561173	k=2.29783		
	M0	90	-11460.38528<br>5	k=1.89262 ω=0.1674<br>2	10858.<br>42	
	M3	94	-11159.402924	k=1.96834<br>p_0=0.33459 ω_0=0.013<br>13<br>p_1=0.39066 ω_1=0.163<br>20<br>p_2=0.27474 ω_2=0.467<br>01		
	M1a	91	-11330.48344<br>5	k=2.11001<br>p_0=0.76997 ω_0=0.120<br>64<br>p_1=0.23003 ω_1=1.000<br>00	0	
	M2a	93	-11330.48344<br>5	k=2.11001<br>p_0=0.76997 ω_0=0.120<br>64		

				$p_1=0.21414$ $\omega_1=1.00000$<br>$p_2=0.01589$ $\omega_2=1.00000$		
	Branch Aves					
	Model A	93	-3535.073475	$\omega_0= 0.04817$ $\omega_1=1$ $\omega_2=1$	0	17 H**<br>75 Y**<br>91 S *
	Null	92	-3535.073475	$\omega_0=0.04817$ $\omega_1=1$ $\omega_2=1$		
	Branch Aves, Reptilia					
	Model A	93	-3531.127951	$\omega_0=0.04026$ $\omega_1=1$ $\omega_2=1$	0	17 H *<br>75 Y**
	Null	92	-3531.127951	$\omega_0=0.04026$ $\omega_1=1$ $\omega_2=1$		
Dkk2	Free-ratio	185	-5720.827745	k=2.46971		
	M0	94	-8127.198797	k=2.45710 $\omega=0.08520$	300.57	
	M3	93	-7976.915947	k=2.50730<br>$p_0=0.34432$ $\omega_0=0.00000$<br>$p_1=0.46238$ $\omega_1=0.07504$<br>$p_2=0.19330$ $\omega_2=0.35827$		
	M1a	95	-8060.246606	k=2.62996<br>$p_0=0.90911$ $\omega_0=0.06621$<br>$p_1=0.09089$ $\omega_1=1.00000$	0	
	M2a	97	-8060.246606	k=2.62996<br>$p_0=0.90911$ $\omega_0=0.06621$<br>$p_1=0.00018$ $\omega_1=1.00000$<br>$p_2=0.09071$ $\omega_2=1.00000$		
	Branch Pholidota					
	Model A	97	-5742.466277	$\omega_0=0.03694$ $\omega_1=1$ $\omega_2=3.81370$	2.18	27 V**
	Null	96	-5743.555860	$\omega_0=0.03688$ $\omega_1=1$ $\omega_2=1$		

Dkk3	Free-ratio	181	-4390.547561	k=3.31134		
	M0	92	-15903.522329	k=2.60461 ω=0.21226	652.71	
	M3	96	-15577.169181	k=2.69349 p_0=0.35315 ω_0=0.03430 p_1=0.46124 ω_1=0.18561 p_2=0.18561 ω_2=0.65364		
	M1a	93	-15728.805722	k=2.83667 p_0=0.77653 ω_0=0.15571 p_1=0.22347 ω_1=1.00000	0	
	M2a	95	-15728.805722	k=2.83666 p_0=0.77653 ω_0=0.15571 p_1=0.20671 ω_1=1.00000 p_2=0.01677 ω_2=1.00000		
	Branch Aves , Reptilia					
	Model A	95	-4346.008306	ω_0=0.08003 ω_1=1 ω_2=1	0	24 R** 41 R** 52 P ** 58 V**
	Null	94	-4346.008306	ω_0=0.08003 ω_1=1 ω_2=1		
Dkk4	Free-ratio	133	-7375.074004	k=2.91533		
	M0	68	-8825.296273	k=2.93346 ω=0.25398	442.00	
	M3	72	-8604.297266	k=3.07716 p_0=0.29356 ω_0=0.01927 p_1=0.40383 ω_1=0.20459 p_2=0.20459 ω_2=0.66251		
	M1a	69	-8663.832199	k=3.30184 p_0=0.67663 ω_0=0.13542	0	

			p_1=0.32337 ω_1=1.00000		
M2a	71	-8663.832199	k=3.30184<br>p_0=0.67663 ω_0=0.13542<br>p_1=0.13305 ω_1=1.00000<br>p_2=0.19032 ω_2=1.00000		
Branch Reptilia					
Model A	71	-7278.405951	ω_0=0.10941 ω_1=1 ω_2=1	0	103 S*<br>105 K*<br>108 Q*
Null	70	-7278.405951	ω_0=0.10941 ω_1=1 ω_2=1		

A

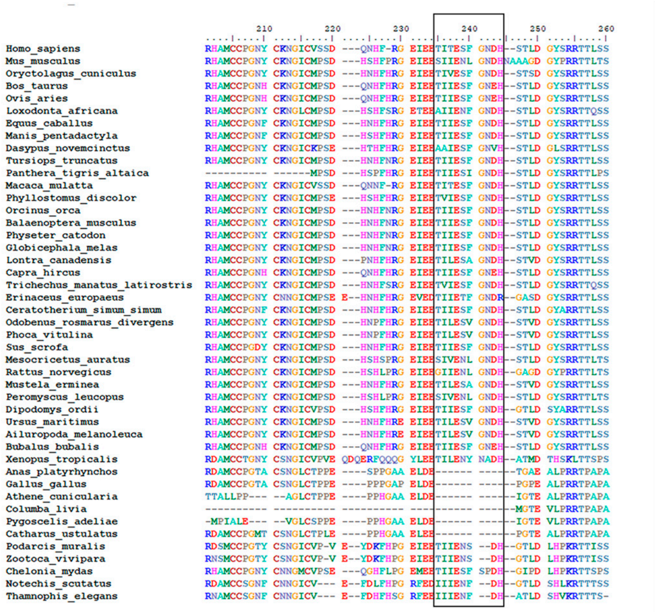

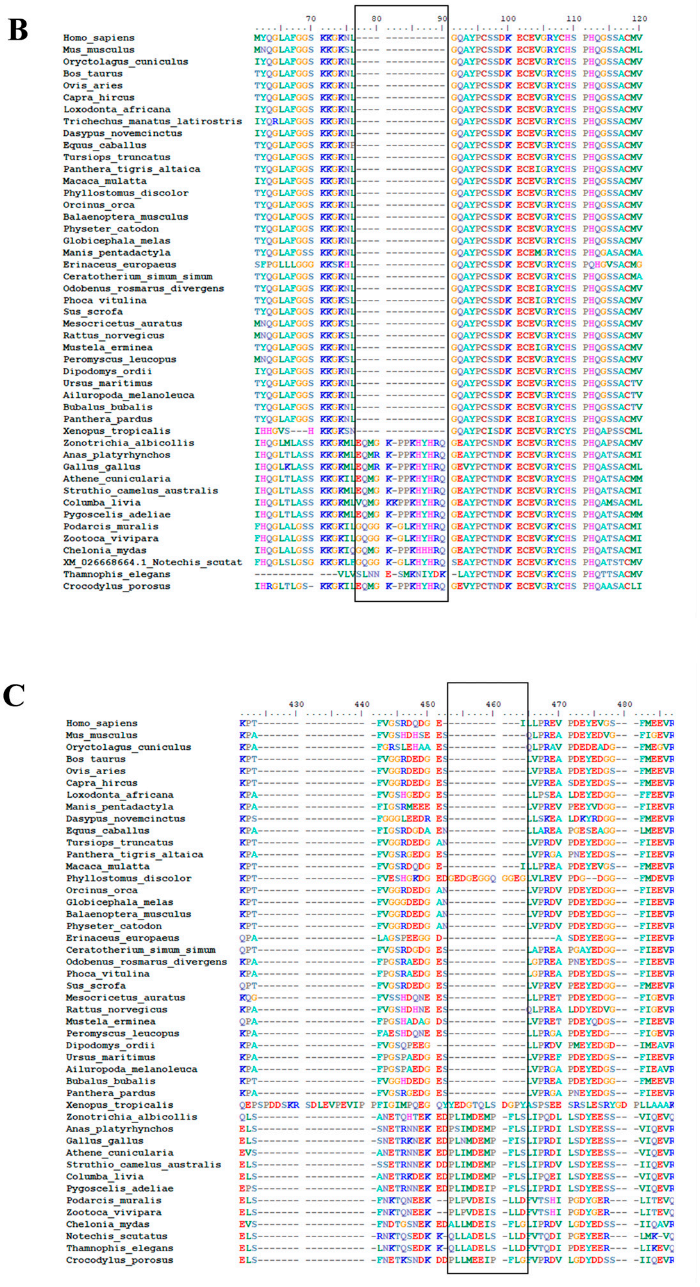

Figure S1. Multiple sequence alignment. (A) In Dkk1, Aves lack 10 amino acids in the middle region, while 10 amino acids are inserted in other vertebrates. (B) In Dkk2, Mammals and Anura lacked 13 amino acids in the middle segment, and 14 amino acids were inserted in Aves and Reptilia. (C) In Dkk3, 12 amino acids were inserted in Xenopus tropicalis and Phyllostomus discolor and Amphibia, 11 amino acids were inserted in Aves and Reptilia.

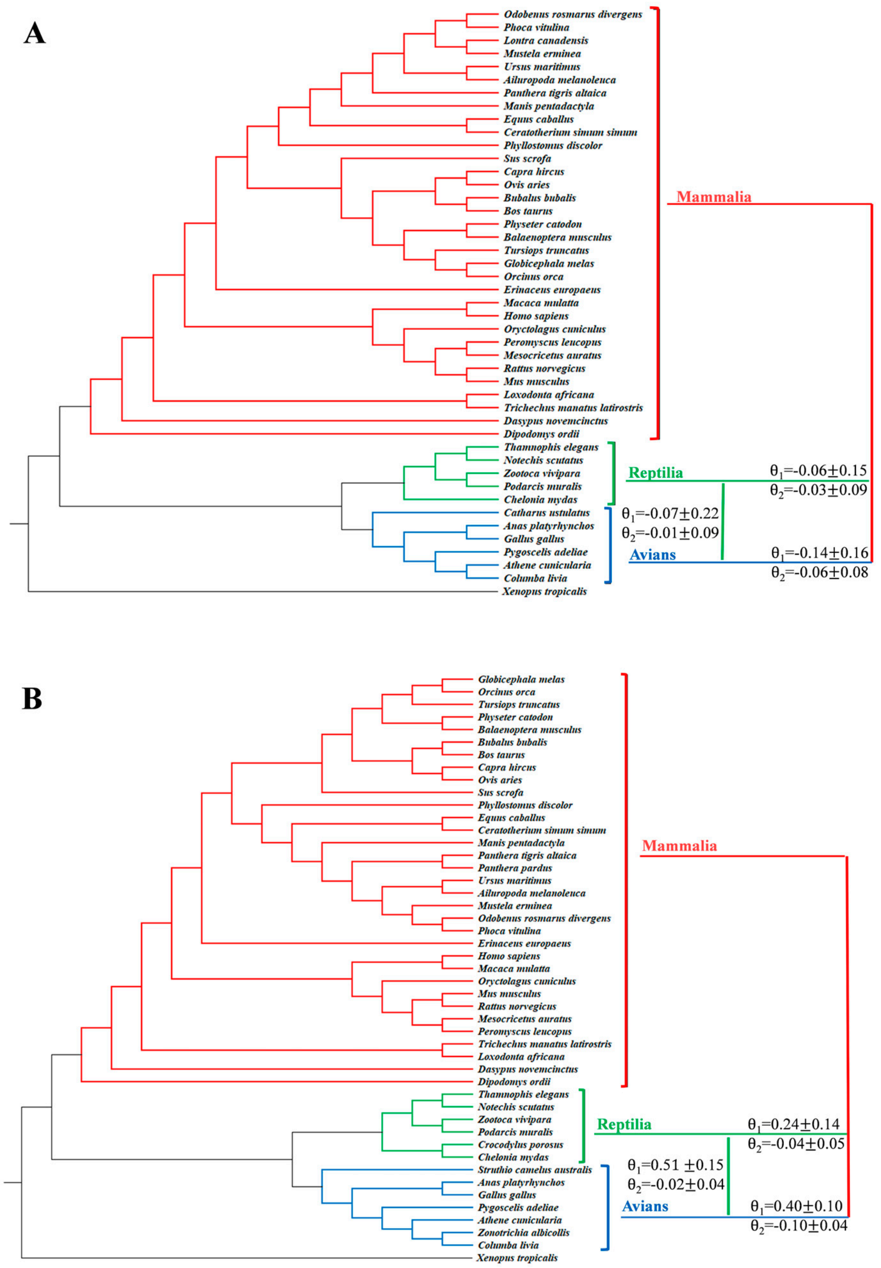
A
Odobenus rosmarus divergens
Phoca vitulina
Lontra canadensis
Mustela erminea
Ursus maritimus
Ailuropoda melanoleuca
Panthera tigris altaica
Manis pentadactyla
Equus caballus
Ceratotherium simum simum
Phyllostomus discolor
Sus scrofa
Capra hircus
Ovis aries
Bubalus bubalis
Bos taurus
Physeter catodon
Balaenoptera musculus
Tursiops truncatus
Globicephala melas
Orcinus orca
Erinaceus europaeus
Macaca mulatta
Homo sapiens
Oryctolagus cuniculus
Peromyscus leucopus
Mesocricetus auratus
Rattus norvegicus
Mus musculus
Loxodonta africana
Trichechus manatus latirostris
Dasypus novemcinctus
Dipodomys ordii
Thamnophis elegans
Notechis scutatus
Zootoca vivipara
Podarcis muralis
Chelonia mydas
Catharus ustulatus
Anas platyrhynchos
Gallus gallus
Pygoscelis adeliae
Athene cunicularia
Columba livia
Xenopus tropicalis
Mammalia
Reptilia
θ_1=-0.06±0.15
θ_2=-0.03±0.09
θ_1=-0.07±0.22
θ_2=-0.01±0.09
Avians
θ_1=-0.14±0.16
θ_2=-0.06±0.08
B
Globicephala melas
Orcinus orca
Tursiops truncatus
Physeter catodon
Balaenoptera musculus
Bubalus bubalis
Bos taurus
Capra hircus
Ovis aries
Sus scrofa
Phyllostomus discolor
Equus caballus
Ceratotherium simum simum
Manis pentadactyla
Panthera tigris altaica
Panthera pardus
Ursus maritimus
Ailuropoda melanoleuca
Mustela erminea
Odobenus rosmarus divergens
Phoca vitulina
Erinaceus europaeus
Homo sapiens
Macaca mulatta
Oryctolagus cuniculus
Mus musculus
Rattus norvegicus
Mesocricetus auratus
Peromyscus leucopus
Trichechus manatus latirostris
Loxodonta africana
Dasypus novemcinctus
Dipodomys ordii
Thamnophis elegans
Notechis scutatus
Zootoca vivipara
Podarcis muralis
Crocodylus porosus
Chelonia mydas
Struthio camelus australis
Anas platyrhynchos
Gallus gallus
Pygoscelis adeliae
Athene cunicularia
Zonotrichia albicollis
Columba livia
Xenopus tropicalis
Mammalia
Reptilia
θ_1=0.24±0.14
θ_2=-0.04±0.05
θ_1=0.51 ±0.15
θ_2=-0.02±0.04
Avians
θ_1=0.40±0.10
θ_2=-0.10±0.04

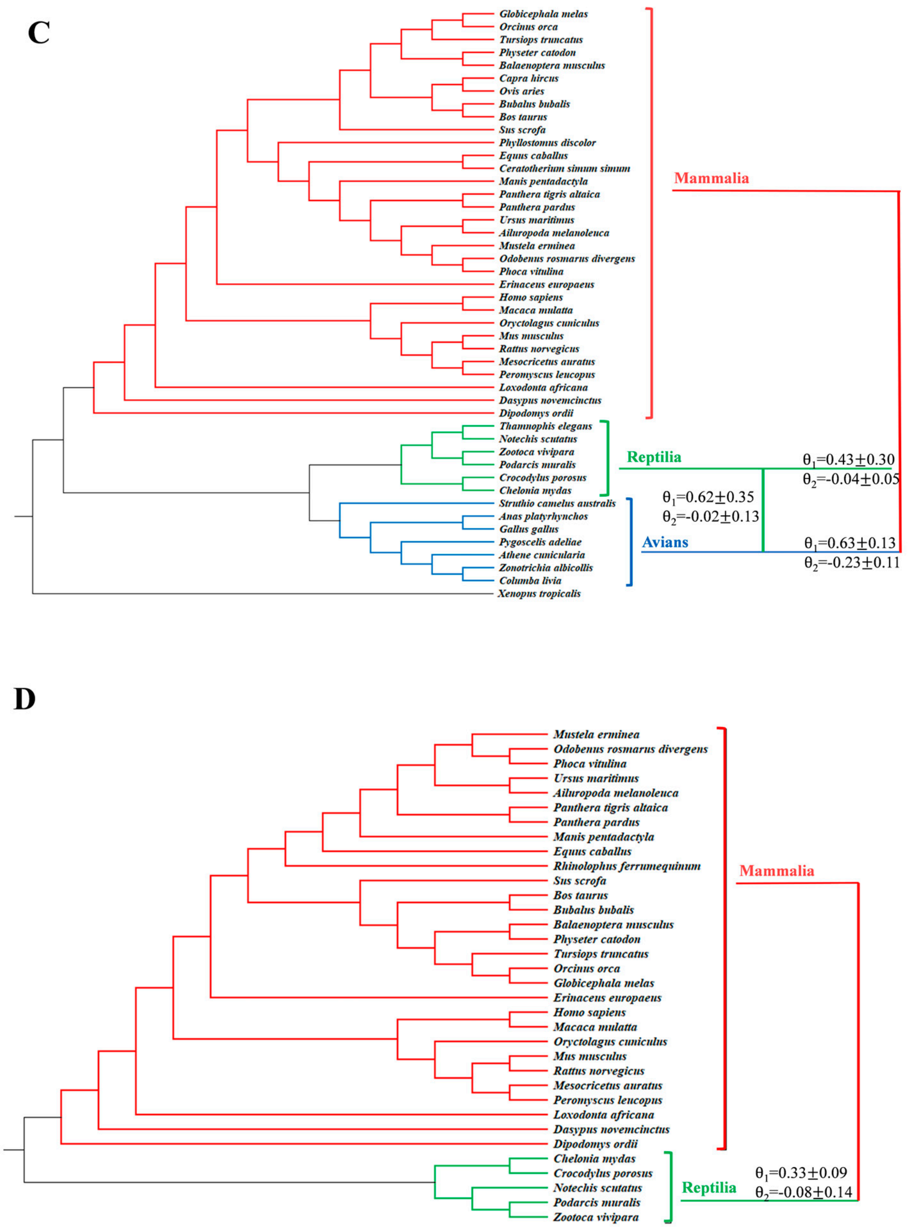

Figure S2 Phylogenetic Tree of Dkk Gene Family. (A) The functional divergene between type I (θI) and type II (θII) of Dkk1 gene were estimated. Between Mammals and Reptilia, Mammals and Aves, Reptilia and Aves. (B) The functional divergene between type I (θI) and type II (θII) of Dkk2 gene were estimated. Between Mammals and Reptilia, Mammals and Aves, Reptilia and Aves. (C) The functional divergene between type I (θI) and type II (θII) of Dkk3 gene were estimated. Between Mammals and Reptilia, Mammals and Aves, Reptilia and Aves. (D) The functional divergene between type I (θI) and type II (θII) of Dkk4 gene were estimated. Between Mammals and Reptilia.

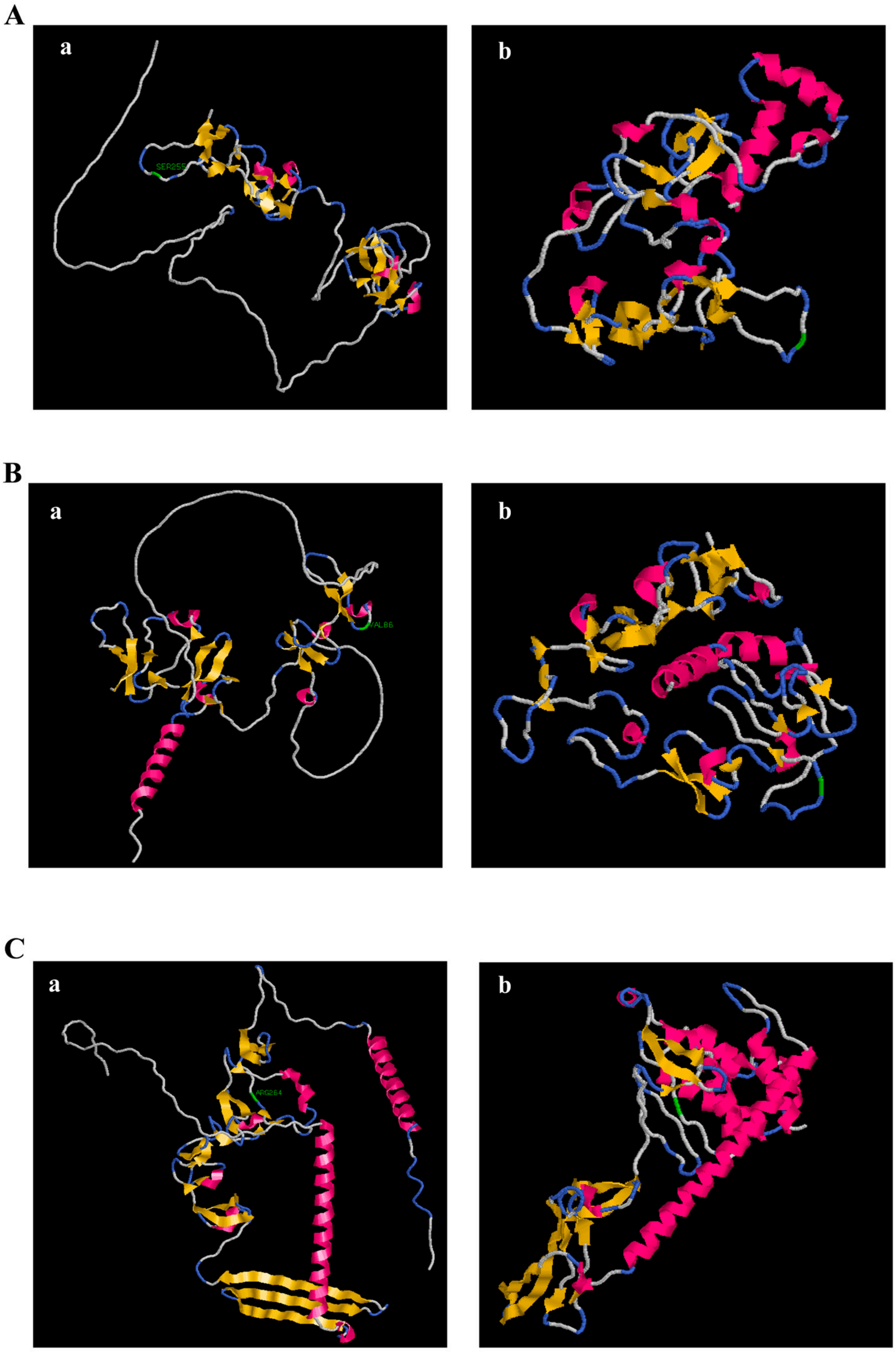
A
a
SER255
b
B
a
VAL86
b
C
a
ARG264
b

D

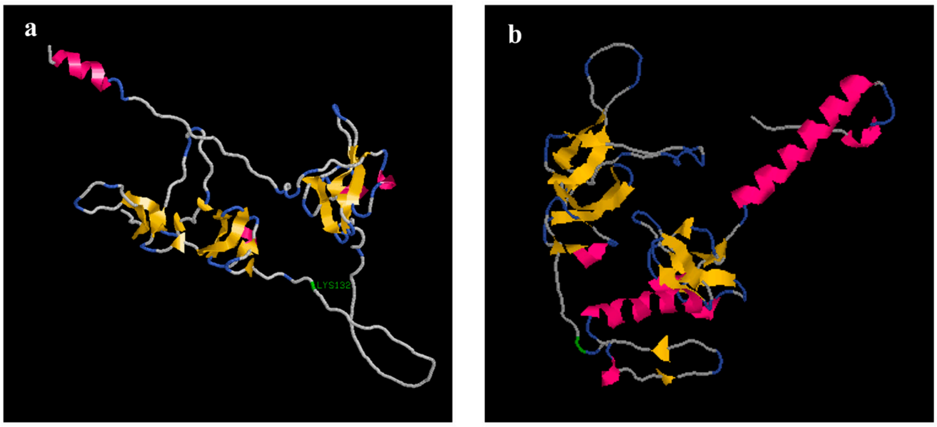

Figure S3. The amino acids under positive selection are mapped onto the 3D structure of the protein. (A) In Dkk1, a is Homo sapiens, the positive selected amino acid (255 S) is shown in green, and the structure is the corner. b is Anas platyrhynchos, the corresponding amino acid (223 P) is shown in green, and the structure is coil. (B) In Dkk2, a is Homo sapiens, the positive selected amino acid (86V) is shown in green, and the structure is the corner. b is Manis pentadactyla, the corresponding amino acid (86M) is shown in green, and the structure is coil. (C) In Dkk3, a is Homo sapiens, the positive selected amino acid (264R) is shown in green, and the structure is the corner. b is Zonotrichia albicollis, the corresponding amino acid (209L) is shown in green, and the structure is no regular coil. (D) In Dkk4, a is Homo sapiens, the positive selection amino acid (132K) is shown in green, and the structure is irregular coil. b is Podarcis muralis, the corresponding amino acid (131Q) is shown in green, and the structure is the corner.
