# Supplementary material for: Molecular Evolution and Protein Structure Variation of Dkk Family
Source: Genes (Basel). 2023 Sep 25;14(10):1863. doi: 10.3390/genes14101863 (PMC10606412; doi:10.3390/genes14101863)
Supplement: Supplementary file 1 [file genes-14-01863-s001.zip › Supplementary material.docx]

**Molecular Evolution and Protein Structure Variation of *Dkk* Family**

Binhong Wen^1^, Husile Gong^2^, Jun Yin^3^, Jianghong Wu^1*^，WenRui Guo^4^

1. College of Animal Science and Technology, Inner Mongolia Minzu University, Tongliao, 028000, China

2. College of Life Science, Inner Mongolia Minzu University, Tongliao, 028000, China

3. College of Animal Science, Inner Mongolia Agricultural University, Hohhot,01001, China

4. College of Veterinary Medicine Inner Mongolia Agricultural University, Hohhot, 010018, China

* Corresponding authors: Jianghong Wu ([wujianghonglong@126.com](mailto:wujianghonglong@126.com), College of Animal Science and Technology, Inner Mongolia Minzu University, Tongliao, 028000, China)

Email addresses

Binhong Wen [wenbinhong99@163.com](mailto:wenbinhong99@163.com)

Husile Gong [huslee@163.com](mailto:huslee@163.com)

Jun Yin [yinjunparis@163.com](mailto:yinjunparis@163.com)

Jianghong Wu [wujianghonglong@126.com](mailto:wujianghonglong@126.com)

WenRui Guo [gwrui@163.com](mailto:gwrui@163.com)

Table S1 Species, scientific names and accession numbers of the *Dkk* gene family

| Gene | Species name | Accession number |
| --- | --- | --- |
| *Dkk1* | Homo sapiens | NM_012242 |
|  | Mus musculus | NM_010051 |
|  | Oryctolagus cuniculus | NM_001082737 |
|  | *Bos taurus* | NM_001205544 |
|  | *Ovis aries* | XM_012102454 |
|  | *Loxodonta africana* | NM_001280880 |
|  | *Equus caballus* | NM_001267802 |
|  | *Manis pentadactyla* | XM_036924093 |
|  | *Dasypus novemcinctus* | XM_004462400 |
|  | *Tursiops truncatus* | XM_004325200 |
|  | *Panthera tigris altaica* | XM_007098121 |
|  | *Macaca mulatta* | NM_001260525 |
|  | *Phyllostomus discolor* | XM_028512165 |
|  | *Orcinus orca* | XM_004286283 |
|  | *Balaenoptera musculus* | XM_036828886 |
|  | *Physeter catodon* | XM_007110600 |
|  | *Globicephala melas* | XM_030864963 |
|  | *Lontra canadensis* | XM_032882999 |
|  | *Capra hircus* | XM_005698161 |
|  | *Trichechus manatus latirostris* | XM_004369892 |
|  | *Erinaceus europaeus* | XM_007520747 |
|  | *Ceratotherium simum simum* | XM_004440005 |
|  | *Odobenus rosmarus divergens* | XM_004408626 |
|  | *Phoca vitulina* | XM_032430367 |
|  | *Mesocricetus auratus* | XM_005063697 |
|  | *Rattus norvegicus* | NM_001106350 |
|  | *Mustela erminea* | XM_032313600 |
|  | *Peromyscus leucopus* | XM_028895210 |
|  | *Dipodomys ordii* | XM_013017131 |
|  | *Ursus maritimus* | XM_008689579 |
|  | *Ailuropoda melanoleuca* | NM_001304925 |
|  | *Bubalus bubalis* | XM_006072352 |
|  | *Panthera pardus* | XM_019430081 |
|  | *Xenopus tropicalis* | NM_001016283 |
|  | *Anas platyrhynchos* | XM_027460718 |
|  | *Gallus gallus* | XM_015278709 |
|  | *Athene cunicularia* | XM_026851768 |
|  | *Columba livia* | XM_005505220 |
|  | *Pygoscelis adeliae* | XM_009332773 |
|  | *Catharus ustulatus* | XM_033066438 |
|  | *Podarcis muralis* | XM_028729659 |
|  | *Zootoca vivipara* | XM_035138222 |
|  | *Chelonia mydas* | XM_007071596 |
|  | *Notechis scutatus* | XM_026680111 |
|  | *Thamnophis elegans* | XM_032231678 |
| *Dkk2* | Homo sapiens | NM_014421 |
|  | Mus musculus | NM_020265 |
|  | Oryctolagus cuniculus | XM_002717198 |
|  | Bos taurus | NM_001082615 |
|  | Ovis aries | XM_004009640 |
|  | *Capra hircus* | XM_005681333 |
|  | *Loxodonta africana* | XM_003410387 |
|  | *Trichechus manatus latirostris* | XM_004380218 |
|  | *Dasypus novemcinctus* | XM_004476199 |
|  | Equus caballus | XM_001503589 |
|  | Tursiops truncatus | XM_019927773 |
|  | *Panthera tigris* altaica | XM_007080813 |
|  | Macaca mulatta | XM_001085254 |
|  | Phyllostomus discolor | XM_028507057 |
|  | Orcinus orca | XM_004269601 |
|  | *Balaenoptera musculus* | XM_036854041 |
|  | *Physeter catodon* | XM_024119509 |
|  | *Globicephala melas* | XM_030864119 |
|  | *Manis pentadactyla* | XM_036920940 |
|  | Erinaceus europaeus | XM_007531232 |
|  | *Ceratotherium simum simum* | XM_004426603 |
|  | *Odobenus rosmarus divergens* | XM_004401893 |
|  | Phoca vitulina | XM_032400962 |
|  | Sus scrofa | XM_003129269 |
|  | Mesocricetus auratus | XM_005081990 |
|  | Rattus norvegicus | NM_001106472 |
|  | *Mustela erminea* | XM_032333084 |
|  | *Peromyscus leucopus* | XM_028856981 |
|  | *Dipodomys ordii* | XM_013023723 |
|  | Ursus maritimus | XM_008689401 |
|  | *Ailuropoda melanoleuca* | XM_002916941 |
|  | Bubalus bubalis | XM_006051404 |
|  | *Panthera pardus* | XM_019416677 |
|  | Xenopus tropicalis | XM_002940290 |
|  | *Zonotrichia albicollis* | XM_005484690 |
|  | *Anas platyrhynchos* | XM_021272669 |
|  | Gallus gallus | XM_420494 |
|  | Athene cunicularia | XM_026848166 |
|  | *Struthio camelus australis* | XM_009689378 |
|  | Columba livia | XM_021283065 |
|  | Pygoscelis adeliae | XM_009327057 |
|  | *Podarcis muralis* | XM_028744214 |
|  | Zootoca vivipara | XM_035113453 |
|  | Chelonia mydas | XM_007054398 |
|  | Notechis scutatus | XM_026668664 |
|  | Thamnophis elegans | XM_032224362 |
|  | *Crocodylus porosus* | XM_019534137 |
| *Dkk3* | *Hydra magnipapillata* | AY332609 |
|  | *Homo sapiens* | NM_001018057 |
|  | *Mus musculus* | NM_001360257 |
|  | *Oryctolagus cuniculus* | XM_008266255 |
|  | *Bos taurus* | NM_001100306 |
|  | *Ovis aries* | XM_027979387 |
|  | *Capra hircus* | XM_018059550 |
|  | *Loxodonta africana* | XM_023550844 |
|  | *Manis pentadactyla* | XM_036918028 |
|  | *Dasypus novemcinctus* | XM_012522734 |
|  | *Equus caballus* | XM_023646049 |
|  | *Tursiops truncatus* | XM_019948202 |
|  | *Panthera tigris altaica* | XM_015537970 |
|  | Macaca mulatta | XM_028832590 |
|  | Phyllostomus discolor | XM_028515585 |
|  | Orcinus orca | XM_004279179 |
|  | Globicephala melas | XM_030831410 |
|  | Balaenoptera musculus | XM_036861902 |
|  | Physeter catodon | XM_024118577 |
|  | Erinaceus europaeus | XM_007521154 |
|  | *Ceratotherium simum simum* | XM_004418440 |
|  | *Odobenus rosmarus divergens* | XM_004394593 |
|  | Phoca vitulina | XM_032427021 |
|  | Sus scrofa | NM_001039749 |
|  | Mesocricetus auratus | XM_013117477 |
|  | Rattus norvegicus | NM_138519 |
|  | *Mustela erminea* | XM_032358081 |
|  | *Peromyscus leucopus* | XM_028875843 |
|  | *Dipodomys ordii* | XM_013028673 |
|  | Ursus maritimus | XM_008711972 |
|  | *Ailuropoda melanoleuca* | XM_002925297 |
|  | Bubalus bubalis | XM_006055228 |
|  | *Panthera pardus* | XM_019456330 |
|  | Xenopus tropicalis | NM_001123476 |
|  | *Zonotrichia albicollis* | XM_005492066 |
|  | *Anas platyrhynchos* | XM_027461217 |
|  | Gallus gallus | NM_205125 |
|  | Athene cunicularia | XM_026850892 |
|  | *Struthio camelus australis* | XM_009687258 |
|  | Columba livia | XM_005499299 |
|  | Pygoscelis adeliae | XM_009334472 |
|  | *Podarcis muralis* | XM_028705427 |
|  | Zootoca vivipara | XM_035119042 |
|  | Chelonia mydas | XM_037899824 |
|  | Notechis scutatus | XM_026664838 |
|  | Thamnophis elegans | XM_032224195 |
|  | *Crocodylus porosus* | XM_019552185 |
| *Dkk4* | *Homo sapiens* | NM_014420 |
|  | *Mus musculus* | NM_145592 |
|  | *Oryctolagus cuniculus* | XM_002720793 |
|  | *Bos taurus* | XM_015460894 |
|  | *Loxodonta africana* | XM_003412504 |
|  | *Manis pentadactyla* | XM_036877486 |
|  | *Dasypus novemcinctus* | XM_004481696 |
|  | *Equus caballus* | XM_001915312 |
|  | *Tursiops truncatus* | XM_033848813 |
|  | *Panthera tigris altaica* | XM_007097034 |
|  | Macaca mulatta | XM_001097758 |
|  | Rhinolophus ferrumequinum | XM_033105033 |
|  | Orcinus orca | XM_004285084 |
|  | Globicephala melas | XM_030829797 |
|  | Balaenoptera musculus | XM_036838881 |
|  | Physeter catodon | XM_007127123 |
|  | Erinaceus europaeus | XM_007522781 |
|  | *Odobenus rosmarus divergens* | XM_004415734 |
|  | Phoca vitulina | XM_032413257 |
|  | Sus scrofa  Mesocricetus auratus  Rattus norvegicus | XM_021077993  XM_005066535  NM_001109332 |
|  | *Peromyscus leucopus* | XM_028887817 |
|  | *Dipodomys ordii* | XM_013024410 |
|  | Ursus maritimus | XM_008706289 |
|  | *Ailuropoda melanoleuca* | XM_019798688 |
|  | Bubalus bubalis | XM_006075711 |
|  | *Panthera pardu* | XM_019440676 |
|  | Xenopus tropicalis | NM_001123476 |
|  | *Podarcis muralis* | XM_028741144 |
|  | Zootoca vivipara | XM_035118651 |
|  | Chelonia mydas | XM_007064183 |
|  | Notechis scutatus | XM_026684761 |
|  | *Crocodylus porosus* | XM_019546036 |

Table.S2 parameters and results of evolutionary model of *Dkk* gene family

| Gene | Model | np | InL | Parameter | 2*Δl* | Positive selection site |
| --- | --- | --- | --- | --- | --- | --- |
| *Dkk1* | Free-ratio | 177 | -3570.561173 | k=2.29783 |  |  |
|  | M0 | 90 | -11460.385285 | k=1.89262 *ω*=0.16742 | 10858.42 |  |
|  | M3 | 94 | -11159.402924 | k=1.96834  p_0_=0.33459 *ω_0_*=0.01313  p_1_=0.39066 *ω_1_*=0.16320  p_2_=0.27474 *ω_2_*=0.46701 |  |  |
|  | M1a | 91 | -11330.483445 | k=2.11001  p_0_=0.76997 *ω_0_*=0.12064  p_1_=0.23003 *ω_1_*=1.00000 | 0 |  |
|  | M2a | 93 | -11330.483445 | k=2.11001  p_0_=0.76997 *ω_0_*=0.12064  p_1_=0.21414 *ω_1_*=1.00000  p_2_=0.01589 *ω_2_*=1.00000 |  |  |
|  | Branch Aves |  |  |  |  |  |
|  | Model A | 93 | -3535.073475 | *ω_0_*= 0.04817 *ω_1_*=1 *ω_2_*=1 | 0 | 17 H**  75 Y**  91 S * |
|  | Null | 92 | -3535.073475 | *ω_0_*=0.04817 *ω_1_*=1 *ω_2_*=1 |  |  |
|  | Branch Aves, Reptilia |  |  |  |  |  |
|  | Model A | 93 | -3531.127951 | *ω_0_*=0.04026 *ω_1_*=1 *ω_2_*=1 | 0 | 17 H *  75 Y** |
|  | Null | 92 | -3531.127951 | *ω_0_*=0.04026 *ω_1_*=1 *ω_2_*=1 |  |  |
| *Dkk2* | Free-ratio | 185 | -5720.827745 | k=2.46971 |  |  |
|  | M0 | 94 | -8127.198797 | k=2.45710 *ω*=0.08520 | 300.57 |  |
|  | M3 | 93 | -7976.915947 | k=2.50730  p_0_=0.34432 *ω_0_*=0.00000  p_1_=0.46238 *ω_1_*=0.07504  p_2_=0.19330 *ω_2_*=0.35827 |  |  |
|  | M1a | 95 | -8060.246606 | k=2.62996  p_0_=0.90911 *ω_0_*=0.06621  p_1_=0.09089 *ω_1_*=1.00000 | 0 |  |
|  | M2a | 97 | -8060.246606 | k=2.62996  p_0_=0.90911 *ω_0_*=0.06621  p_1_=0.00018 *ω_1_*=1.00000  p_2_=0.09071 *ω_2_*=1.00000 |  |  |
|  | Branch Pholidota |  |  |  |  |  |
|  | Model A | 97 | -5742.466277 | *ω_0_*=0.03694 *ω_1_*=1 *ω_2_*=3.81370 | 2.18 | 27 V** |
|  | Null | 96 | -5743.555860 | *ω_0_*=0.03688 *ω_1_*=1 *ω_2_*=1 |  |  |
| *Dkk3* | Free-ratio | 181 | -4390.547561 | k=3.31134 |  |  |
|  | M0 | 92 | -15903.522329 | k=2.60461 *ω*=0.21226 | 652.71 |  |
|  | M3 | 96 | -15577.169181 | k=2.69349  p_0_=0.35315 *ω_0_*=0.03430  p_1_=0.46124 *ω_1_*=0.18561  p_2_=0.18561 *ω_2_*=0.65364 |  |  |
|  | M1a | 93 | -15728.805722 | k=2.83667  p_0_=0.77653 *ω_0_*=0.15571  p_1_=0.22347 *ω_1_*=1.00000 | 0 |  |
|  | M2a | 95 | -15728.805722 | k=2.83666  p_0_=0.77653 *ω_0_*=0.15571  p_1_=0.20671 *ω_1_*=1.00000  p_2_=0.01677 *ω_2_*=1.00000 |  |  |
|  | Branch Aves ,Reptilia |  |  |  |  |  |
|  | Model A | 95 | -4346.008306 | *ω_0_*=0.08003 *ω_1_*=1 *ω_2_*=1 | 0 | 24 R**  41 R**  52 P **  58 V** |
|  | Null | 94 | -4346.008306 | *ω_0_*=0.08003 *ω_1_*=1 *ω_2_*=1 |  |  |
| *Dkk4* | Free-ratio | 133 | -7375.074004 | k=2.91533 |  |  |
|  | M0 | 68 | -8825.296273 | k=2.93346 *ω*=0.25398 | 442.00 |  |
|  | M3 | 72 | -8604.297266 | k=3.07716  p_0_=0.29356 *ω_0_*=0.01927  p_1_=0.40383 *ω_1_*=0.20459  p_2_=0.20459 *ω_2_*=0.66251 |  |  |
|  | M1a | 69 | -8663.832199 | k=3.30184  p_0_=0.67663 *ω_0_*=0.13542  p_1_=0.32337 *ω_1_*=1.00000 | 0 |  |
|  | M2a | 71 | -8663.832199 | k=3.30184  p_0_=0.67663 *ω_0_*=0.13542  p_1_=0.13305 *ω_1_*=1.00000  p_2_=0.19032 *ω_2_*=1.00000 |  |  |
|  | Branch Reptilia |  |  |  |  |  |
|  | Model A | 71 | -7278.405951 | *ω_0_*=0.10941 *ω_1_*=1 *ω_2_*=1 | 0 | 103 S*  105 K*  108 Q* |
|  | Null | 70 | -7278.405951 | *ω_0_*=0.10941 *ω_1_*=1 *ω_2_*=1 |  |  |

| 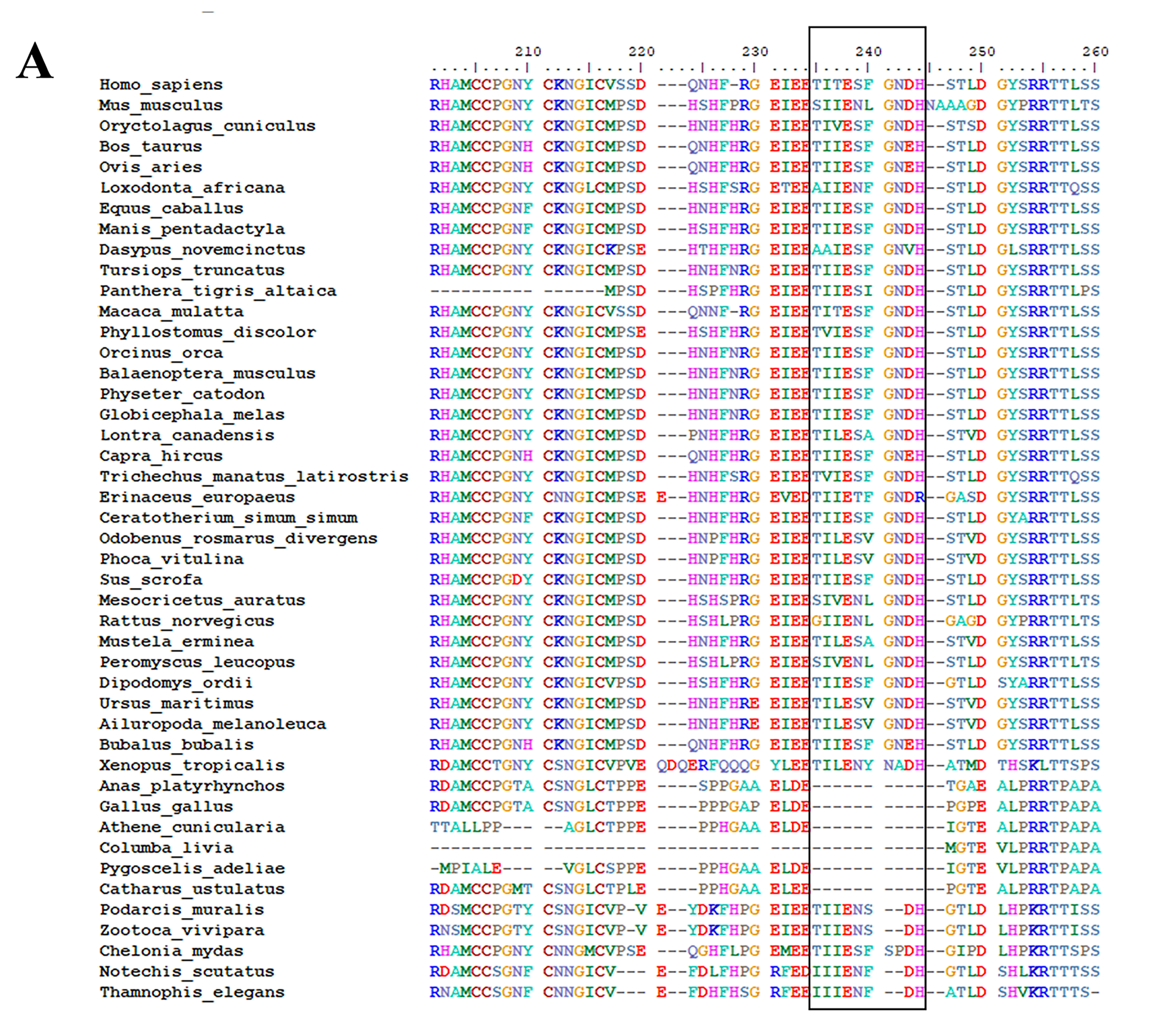 |
| --- |
| 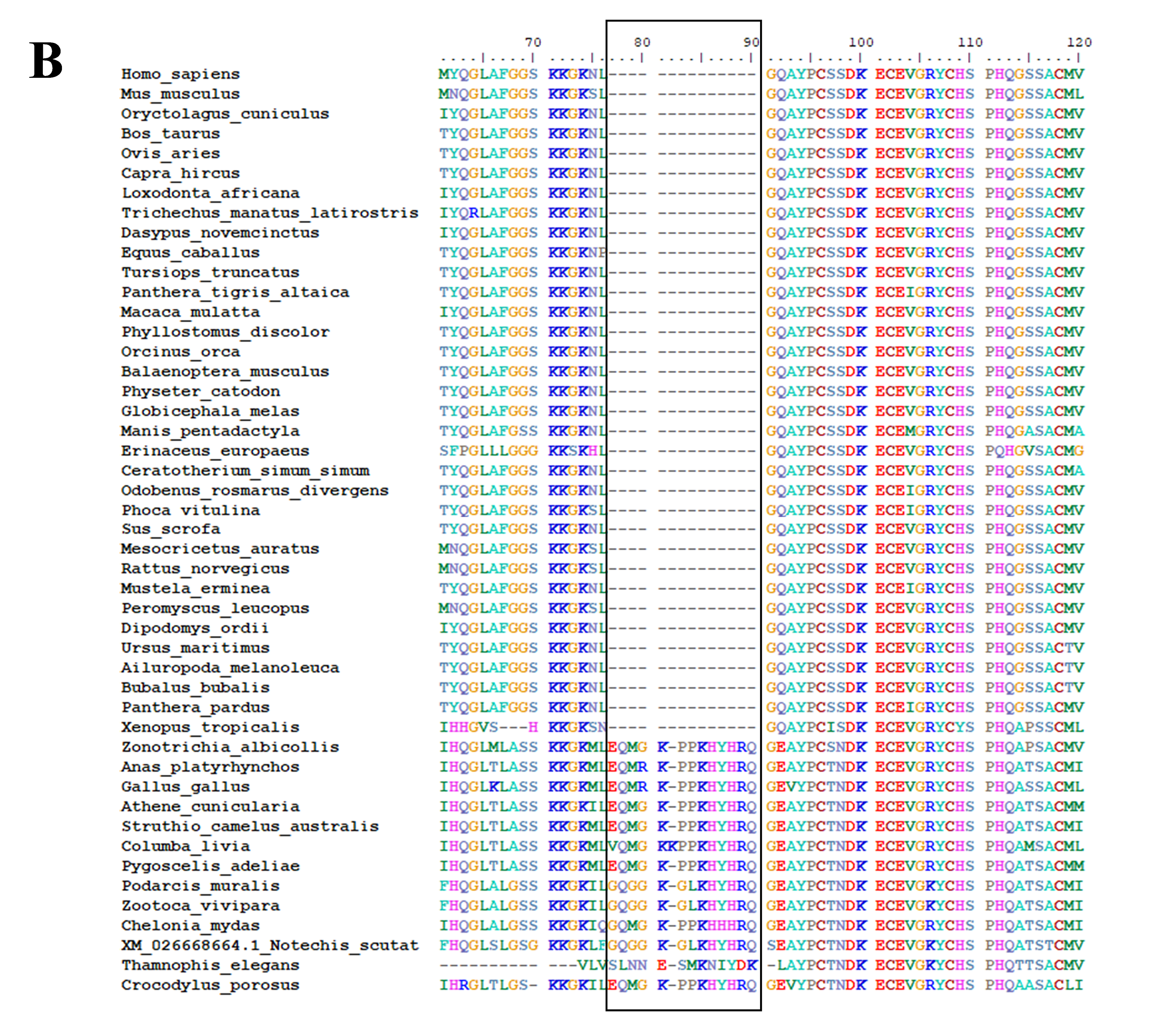 |
| 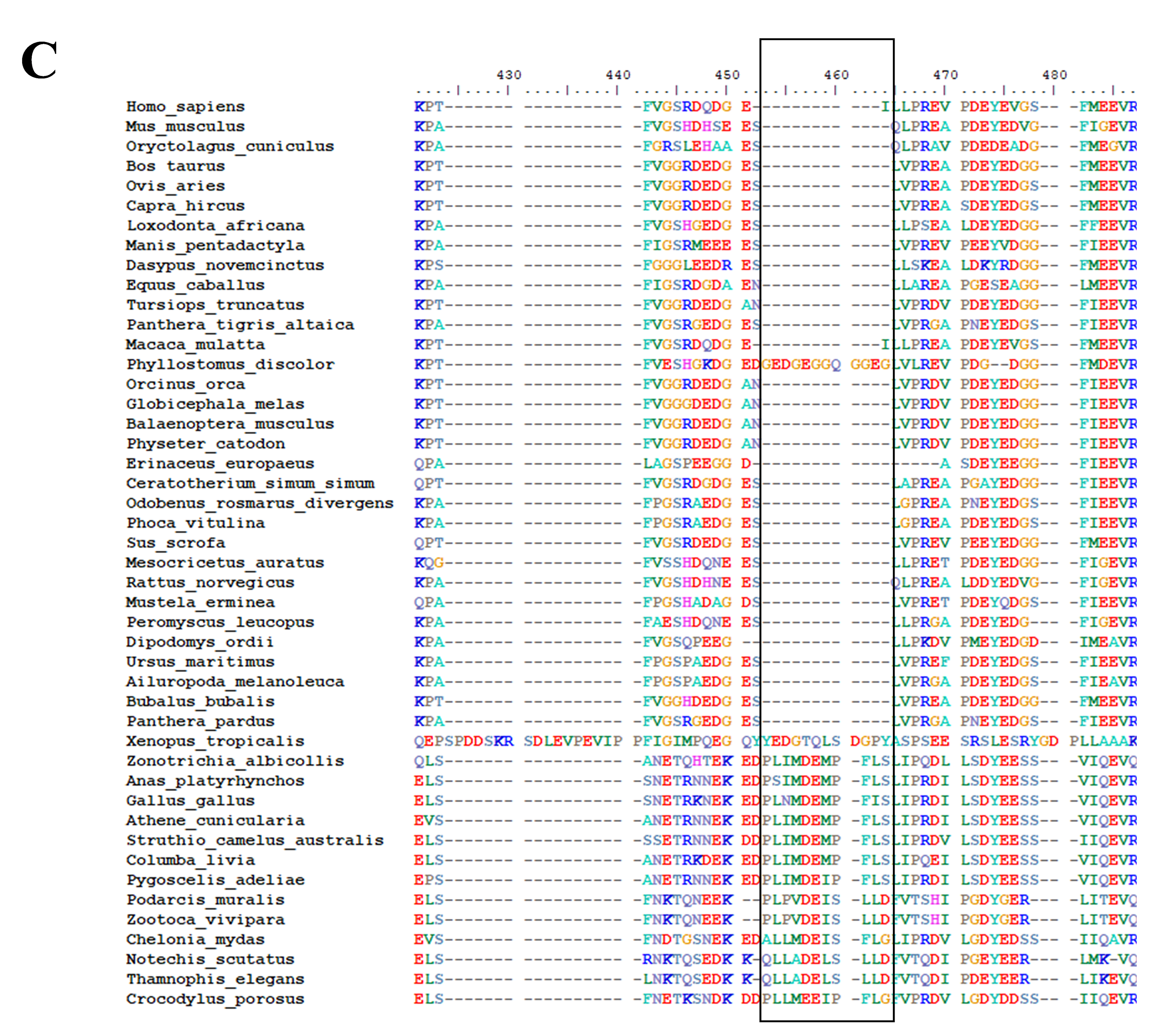 |
| Fig. S1. Multiple sequence alignment. (A) In Dkk1, Aves lack 10 amino acids in the middle region, while 10 amino acids are inserted in other vertebrates. (B) In Dkk2, Mammals and Anura lacked 13 amino acids in the middle segment, and 14 amino acids were inserted in Aves and Reptilia. (C) In Dkk3, 12 amino acids were inserted in Xenopus tropicalis and Phyllostomus discolor and Amphibia, 11 amino acids were inserted in Aves and Reptilia. |

|  |
| --- |
|  |
|  |
|  |
| Fig.S2 Phylogenetic Tree of Dkk Gene Family. (A) The functional divergene between type I (θI) and type II (θII) of Dkk1 gene were estimated. Between Mammals and Reptilia, Mammals and Aves, Reptilia and Aves. (B) The functional divergene between type I (θI) and type II (θII) of Dkk2 gene were estimated. Between Mammals and Reptilia, Mammals and Aves, Reptilia and Aves. (C) The functional divergene between type I (θI) and type II (θII) of Dkk3 gene were estimated. Between Mammals and Reptilia, Mammals and Aves, Reptilia and Aves. (D) The functional divergene between type I (θI) and type II (θII) of Dkk4 gene were estimated. Between Mammals and Reptilia. |


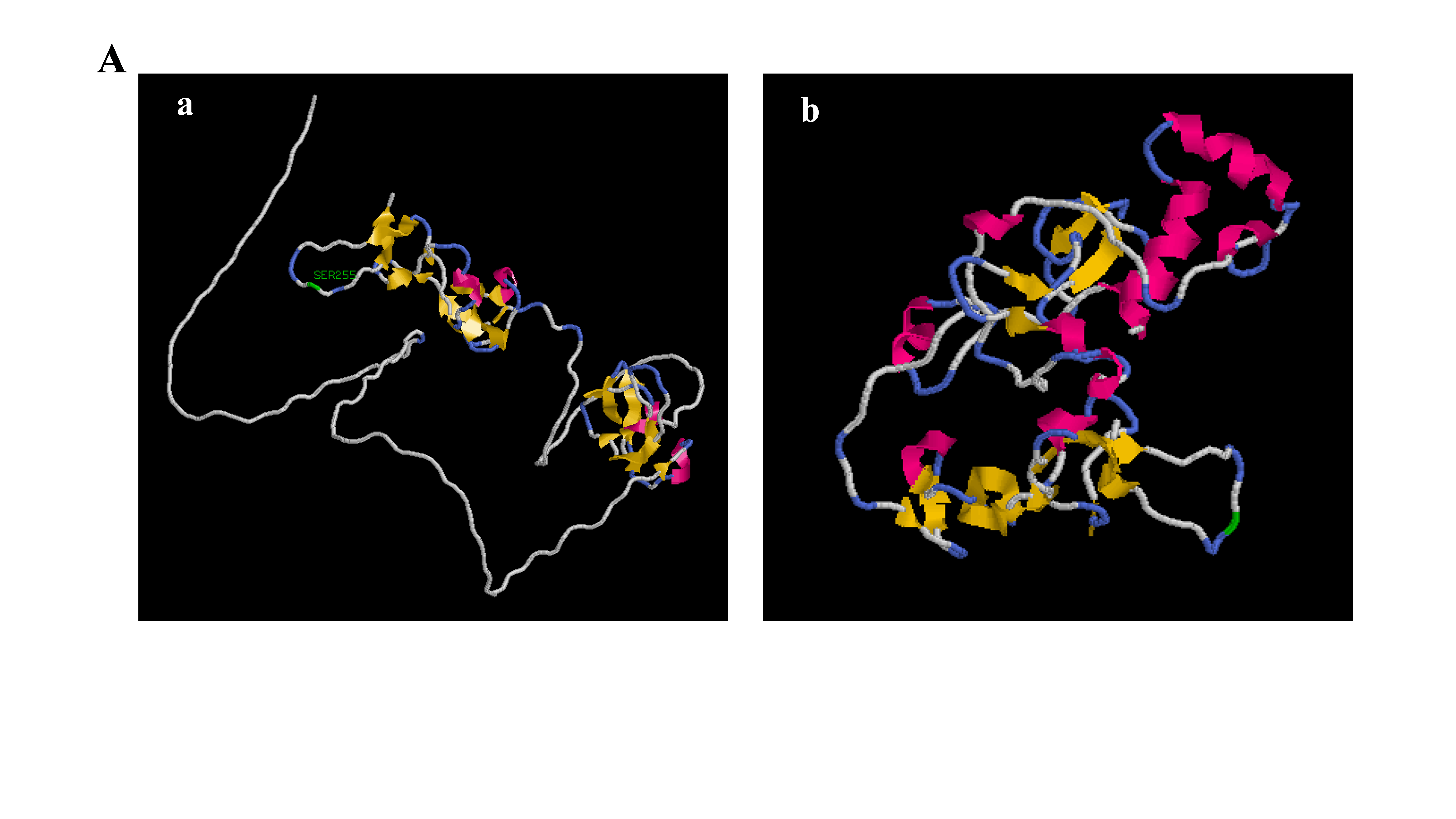


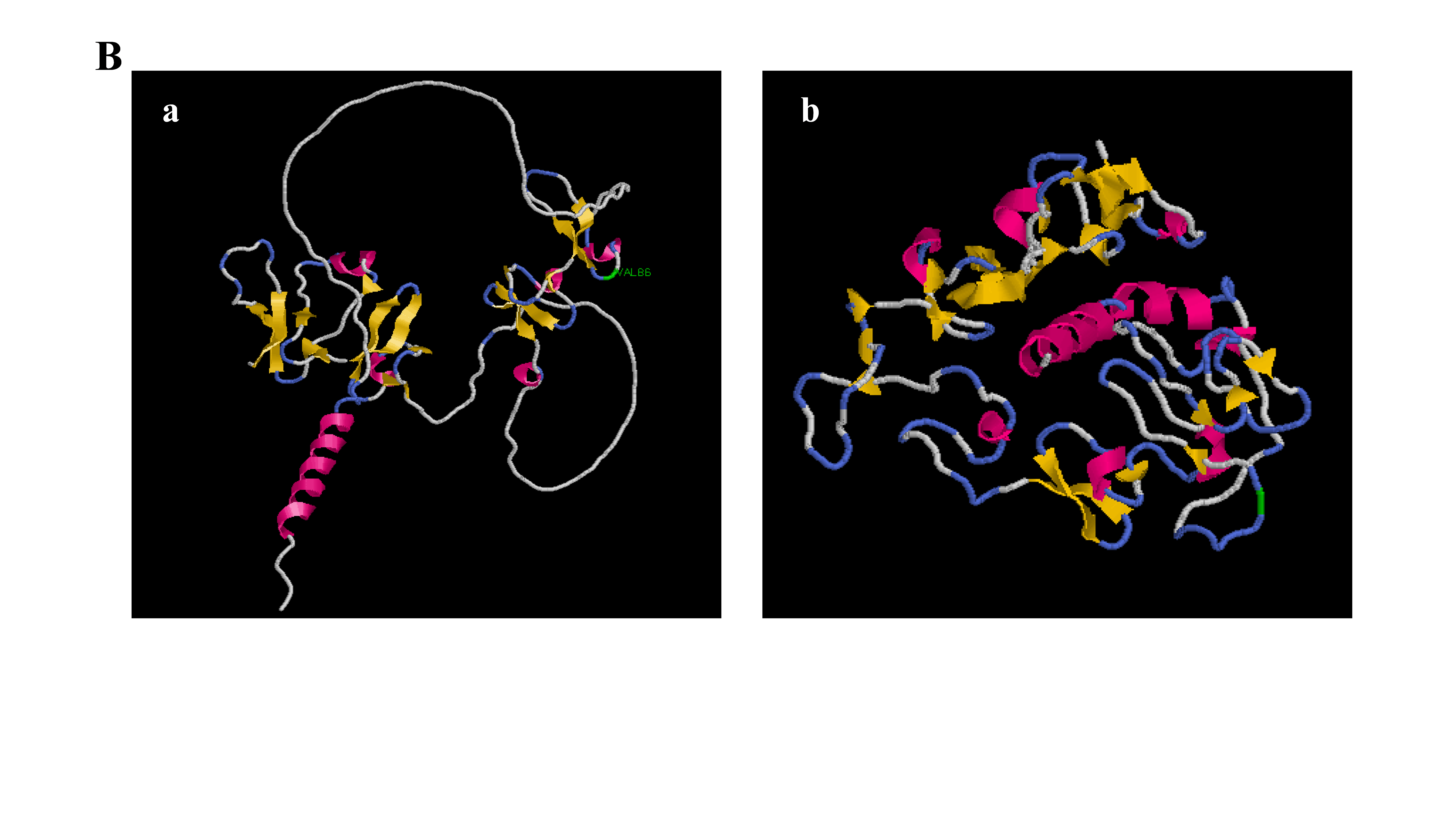


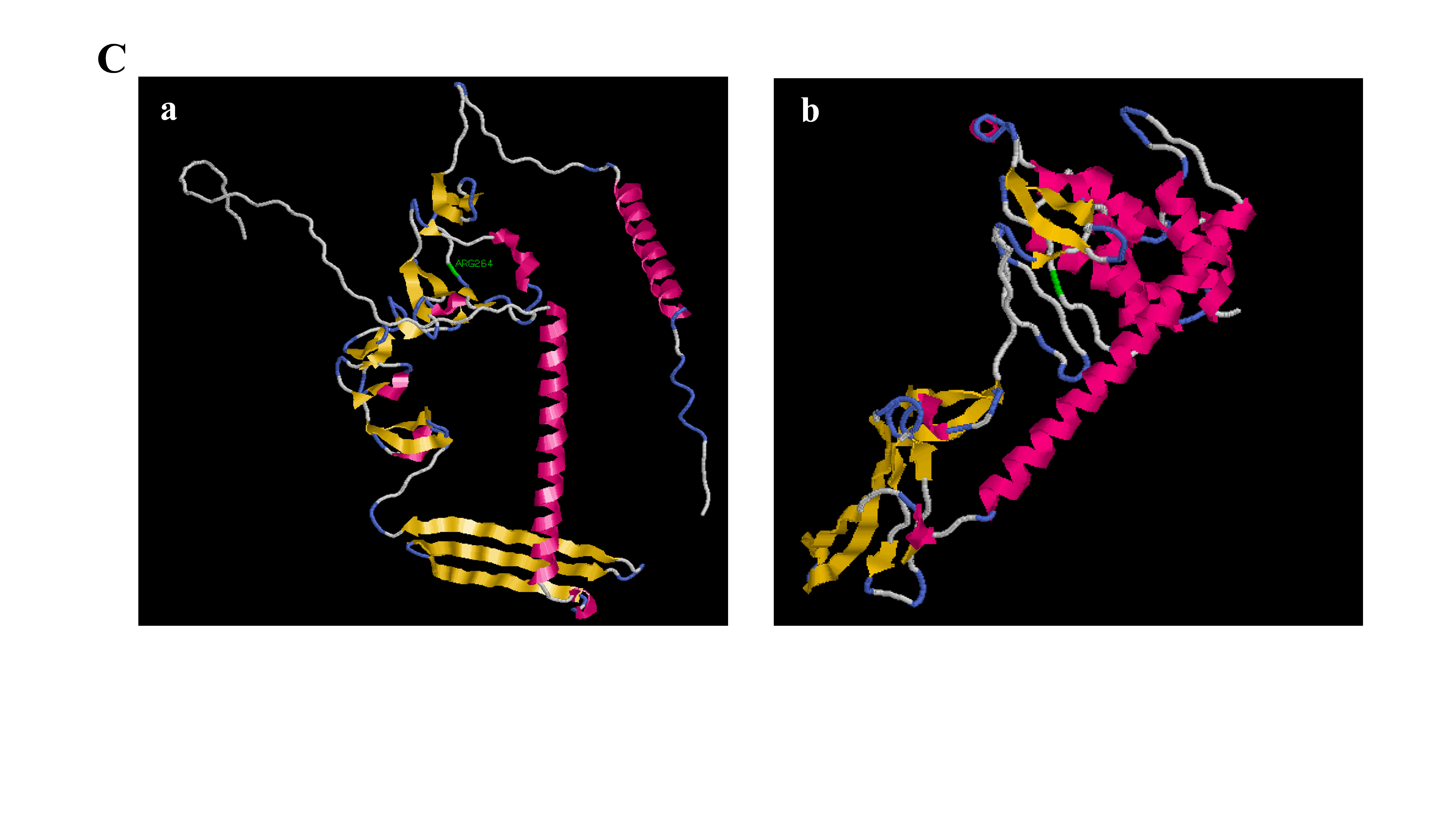


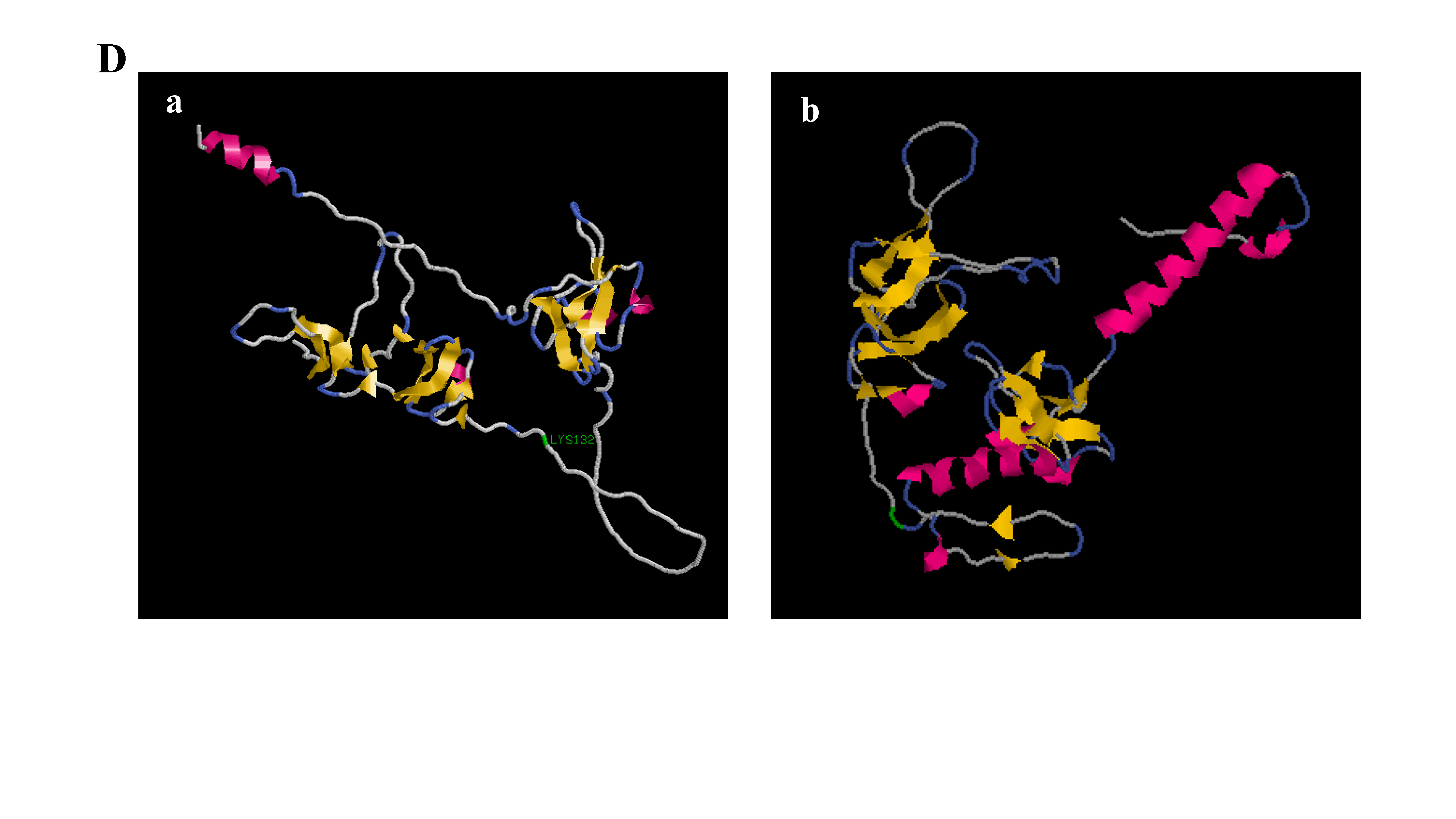


Figure S3. The amino acids under positive selection are mapped onto the 3D structure of the protein. (A) In Dkk1, a is Homo sapiens, the positive selected amino acid (255 S) is shown in green, and the structure is the corner. b is Anas platyrhynchos, the corresponding amino acid (223 P) is shown in green, and the structure is coil. (B) In Dkk2, a is Homo sapiens, the positive selected amino acid (86V) is shown in green, and the structure is the corner. b is Manis pentadactyla, the corresponding amino acid (86M) is shown in green, and the structure is coil. (C) In Dkk3, a is Homo sapiens, the positive selected amino acid (264R) is shown in green, and the structure is the corner. b is Zonotrichia albicollis, the corresponding amino acid (209L) is shown in green, and the structure is no regular coil. (D) In Dkk4, a is Homo sapiens, the positive selection amino acid (132K) is shown in green, and the structure is irregular coil. b is Podarcis muralis, the corresponding amino acid (131Q) is shown in green, and the structure is the corner.
